# Natural variation and CRISPR/Cas9‐mediated mutation in *GmPRR37* affect photoperiodic flowering and contribute to regional adaptation of soybean

**DOI:** 10.1111/pbi.13346

**Published:** 2020-02-13

**Authors:** Liwei Wang, Shi Sun, Tingting Wu, Luping Liu, Xuegang Sun, Yupeng Cai, Jicun Li, Hongchang Jia, Shan Yuan, Li Chen, Bingjun Jiang, Cunxiang Wu, Wensheng Hou, Tianfu Han

**Affiliations:** ^1^ Ministry of Agriculture and Rural Affairs Key Laboratory of Soybean Biology (Beijing) Institute of Crop Sciences Chinese Academy of Agricultural Sciences Beijing China; ^2^ Jining Academy of Agricultural Sciences Jining Shandong China

**Keywords:** soybean (*Glycine max* (L.) Merr.), flowering time, QTL, *GmPRR37*, CRISPR/Cas9, adaptation

## Abstract

Flowering time is a critical determinant of the geographic distribution and regional adaptability of soybean (*Glycine max*) and is strongly regulated by photoperiod and temperature. In this study, quantitative trait locus (QTL) mapping and subsequent candidate gene analysis revealed that *GmPRR37*, encoding a pseudo‐response regulator protein, is responsible for the major QTL *qFT12‐2*, which was identified from a population of 308 recombinant inbred lines (RILs) derived from a cross between a very late‐flowering soybean cultivar, ‘Zigongdongdou (ZGDD)’, and an extremely early‐flowering cultivar, ‘Heihe27 (HH27)’, in multiple environments. Comparative analysis of parental sequencing data confirmed that HH27 contains a non‐sense mutation that causes the loss of the CCT domain in the GmPRR37 protein. CRISPR/Cas9‐induced *Gmprr37*‐ZGDD mutants in soybean exhibited early flowering under natural long‐day (NLD) conditions. Overexpression of *GmPRR37* significantly delayed the flowering of transgenic soybean plants compared with wild‐type under long photoperiod conditions. In addition, both the knockout and overexpression of *GmPRR37* in soybean showed no significant phenotypic alterations in flowering time under short‐day (SD) conditions. Furthermore, *GmPRR37* down‐regulated the expression of the flowering‐promoting *FT* homologues *GmFT2a* and *GmFT5a*, and up‐regulated flowering‐inhibiting *FT* homologue *GmFT1a* expression under long‐day (LD) conditions. We analysed haplotypes of *GmPRR37* among 180 cultivars collected across China and found natural *Gmprr37* mutants flower earlier and enable soybean to be cultivated at higher latitudes. This study demonstrates that *GmPRR37* controls soybean photoperiodic flowering and provides opportunities to breed optimized cultivars with adaptation to specific regions and farming systems.

## Introduction

The transition from the vegetative to reproductive phase is a key developmental switch in flowering plants (Blumel *et al.*, 2015). Activation of the transition to flowering depends on a complex network with (epi‐) genetic factors and environmental stimuli (Blumel *et al.*, 2015; Mouradov *et al.*, 2002). Timing of flowering is critical for adaptability, productivity and quality of seed crops. Thus, understanding the molecular mechanisms underlying flowering is crucial for crop genetic improvement.

Soybean (*Glycine max* (L.) Merr.) is a facultative short‐day (SD) plant and is cultivated worldwide in a broad range of latitudes, although each cultivar is restricted to a relatively narrow latitude range (Watanabe *et al.*, 2012). Photoperiod sensitivity is one of the key determinants for soybean flowering time and is regulated by the interaction between endogenous circadian rhythm and exogenous day length, which varies at different geographical latitudes. Successful identification of major genes and quantitative trait loci (QTL) underlying flowering time is a prerequisite for understanding the soybean photoperiod flowering pathway.

A large number of QTLs in different linkage groups [e.g. first flower‐104, pod maturity beginning (R7)‐5, pod maturity (R8)‐178 QTL] have been identified to be involved in flowering and maturity and are catalogued in SoyBase (http://soybase.org/). Among these, 11 have been confirmed as major effect QTLs that control the time to flowering and maturity in soybean: *E1* and *E2* (Bernard, 1971), *E3* (Buzzell, 1971), *E4* (Buzzell and Voldeng, 1980), *E6* (Bonato and Vello, 1999), *E7* (Cober and Voldeng, 2001), *E8* (Cober *et al.*, 2010), *E9* (Kong *et al.*, 2014), *E10* (Samanfar *et al.*, 2017), *qDTF‐J1* (Takeshima *et al.*, 2016) and *J* (Ray *et al.*, 1995). The genes underlying QTLs *E1*‐*E4*, *E9*, *E10*, *qDTF‐J1* and *J* have been identified, and their functions in the photoperiod control of flowering have been characterized (Kong *et al.*, 2010; Liu *et al.*, 2008; Lu *et al.*, 2017; Samanfar *et al.*, 2017; Sun *et al.*, 2011; Takeshima *et al.*, 2016; Watanabe *et al.*, 2009; Watanabe *et al.*, 2011; Xia *et al.*, 2012; Yue *et al.*, 2017; Zhao *et al.*, 2016). Despite these progresses, bioinformatics analysis has revealed that there were 333 (Watanabe *et al.*, 2012) to 491 (Jung *et al.*, 2012) orthologs of Arabidopsis flowering‐time genes in soybean. Thus, the molecular cloning of genes associated with flowering in soybean has lagged behind that in the model plant Arabidopsis.

In Arabidopsis, photoperiodic flowering relies on circadian clock (Greenham and McClung, 2015). Circadian clocks, which are endogenous mechanisms for keeping time, allow organisms to coordinate biological processes with the time of day and thus provide an adaptive advantage (Bendix *et al.*, 2015; Farre and Liu, 2013). Pseudo‐response regulators (PRRs) are key components of circadian networks in Arabidopsis (Farre *et al.*, 2005; Farre and Liu, 2013; Nakamichi *et al.*, 2012; Perales and Mas, 2007; Wang *et al.*, 2013) and are defined as proteins containing two conserved domains: a N‐terminal response‐regulator receiver domain and a C‐terminal CCT (CONSTANS, CO‐like, and TOC1) domain. Arabidopsis has five PRR family members (*APRR1/TOC1*, *APRR3*, *APRR5*, *APRR7* and *APRR9*). Genetic analysis has revealed that *APRRs* are involved in the regulation of photoperiodic flowering in Arabidopsis (Murakami *et al.*, 2004; Nakamichi *et al.*, 2005). Phylogenetic analyses indicate that the common ancestor of plants possessed homologues of three PRR groups (TOC1, PRR7/3 and PRR5/9) in its genome (Farre and Liu, 2013; Satbhai *et al.*, 2011). Recent studies have demonstrated that PRRs are functionally conserved among plants. For example, several *PRR37* genes showing high sequence similarity to both *PRR7* and *PRR3*, such as *Ppd‐H1* in barley (*Hordeum vulgare*), *Ppd‐D1* in wheat (*Triticum aestivum* L.), *SbPRR37* in sorghum (*Sorghum bicolor* (L.) Moench) and *OsPRR37* in rice (*Oryza sativa* L.), have been shown to regulate photoperiod flowering in crops (Beales *et al.*, 2007; Gao *et al.*, 2014; Koo *et al.*, 2013; Liu *et al.*, 2018a; Murphy *et al.*, 2011; Turner *et al.*, 2005; Yan *et al.*, 2013). In soybean, two genes homologous to *PRR3* (*GmPRR3A* and *GmPRR3B*) are the most likely candidates responsible for two QTLs controlling growth period (Li *et al.*, 2019a). However, the molecular identification, functional characterization and mechanism of *PRR3/7* in soybean have remained elusive.

The objective of this study was to identify QTLs regulating soybean flowering time using a population of 308 recombinant inbred lines (RILs) derived from a cross between a very late‐flowering soybean cultivar, ‘Zigongdongdou (ZGDD)’, and an extremely early‐flowering soybean cultivar, ‘Heihe27 (HH27)’. Through map‐based cloning and candidate gene analysis, we found that *GmPRR37* is responsible for one of the major effect QTLs we identified, *qFT12‐2*. HH27 contains a non‐sense mutation in the CCT domain of the protein encoded by *GmPRR37*. CRISPR/Cas9‐induced *Gmprr37*‐ZGDD mutants exhibit early flowering under natural long‐day (NLD) conditions, while no significant phenotypic alterations in flowering time were observed under SD conditions. Overexpression of the functional ZGDD *GmPRR37* gene in the soybean cultivar Jack significantly delayed flowering time under long‐day (LD) and NLD conditions, but had little effect on flowering time under SD conditions. These results suggest that *GmPRR37* functions as a suppressor in LD‐dependent flowering pathway in soybean. We genotyped *GmPRR37* in 180 cultivars from diverse soybean growing areas across a wide geographic range in China and demonstrated that loss‐of‐function of *GmPRR37* has contributed to the cultivation of soybean in higher latitude regions.

## Results

### Phenotypic analysis of flowering time in the RIL population

The flowering time of 308 RILs and the parents HH27 and ZGDD were determined in Beijing 2016 and 2017, in Xinxiang 2016 and 2017, in Sanya 2016 and 2017, in Jining 2016 and in Xiangtan 2017. Each location‐year combination was defined as an experimental environment, and these eight environments were named BJ16, BJ17, XX16, XX17, SY16, SY17, JN16 and XT17, respectively. There were highly significant differences (*P* < 0.01) in flowering time between the two parents under all environments, with ZGDD flowering much later than HH27. Among the RILs, there was wide variation in flowering time across eight environments, and obvious bi‐directional transgressive segregation in all environments except SY16 and BJ17, suggesting polygenic quantitative genetic control (Figure S1, Table S1).

### Genotyping of the RIL population based on 2b‐RAD and genetic map construction

We used 2b‐RAD to genotype the RIL population. DNA was digested with *Bsa*XI or *Fal*I and sequenced with the Illumina Hiseq X ten platform. For *Bsa*XI‐digested DNA, the sequencing depth was 44× for HH27, 43× for ZGDD and 15× for the 308 RILs (Table S2). For *Fal*I‐digested DNA, the sequencing depth was 93× for HH27, 90× for ZGDD and 27× for the 308 RILs (Table S3). The sequencing depth detail for each RIL is shown in Table S2 and S3. After genotyping, 7,123 polymorphic single‐nucleotide polymorphism (SNP) markers were identified and used for linkage map construction. The final molecular linkage map consisted of 3,454 markers on 20 linkage groups that spanned 2208.16 cM with an average distance of 0.64 cM between adjacent markers (Figure S2, Table S4).

### QTL analysis of flowering time in multiple environments

Based on the high‐density genetic map, a total of 69 flowering‐time QTLs spread over 10 soybean chromosomes were identified through single‐environment QTL analysis (Table S5). Of these QTLs, nine were identified across multiple environments, and six were identified in a single environment (Table S5). The nine stable QTLs detected across multiple environments may explain the genetic basis of soybean flowering regulation, and we mainly focused on these QTLs in subsequent analysis. Comparative analysis showed that well‐characterized genes (*E1*, *E2*, *FT5a*, *FT2a* and *E3*) underlying flowering time in soybean were located within the genomic regions of *qFT6*, *qFT10*, *qFT16‐1*, *qFT16‐2* and *qFT19* (Table S5). *qFT11* was identified in more than five environments and using BLUP values, but it only accounted for 2.26‐5.44% of the phenotypic variation. The major effect QTL *qFT12‐2* was confirmed across seven environments and using BLUP values, with an average logarithm of the odds (LOD) score ranging from 5.37 to 40.11. Furthermore, *qFT12‐2* explained more than 10% of the phenotypic variation in three environments. Based on these results, we chose the major QTL *qFT12‐2* for further analysis.

### Glyma.12G073900 is the candidate gene for qFT12‐2

**Figure 1:**
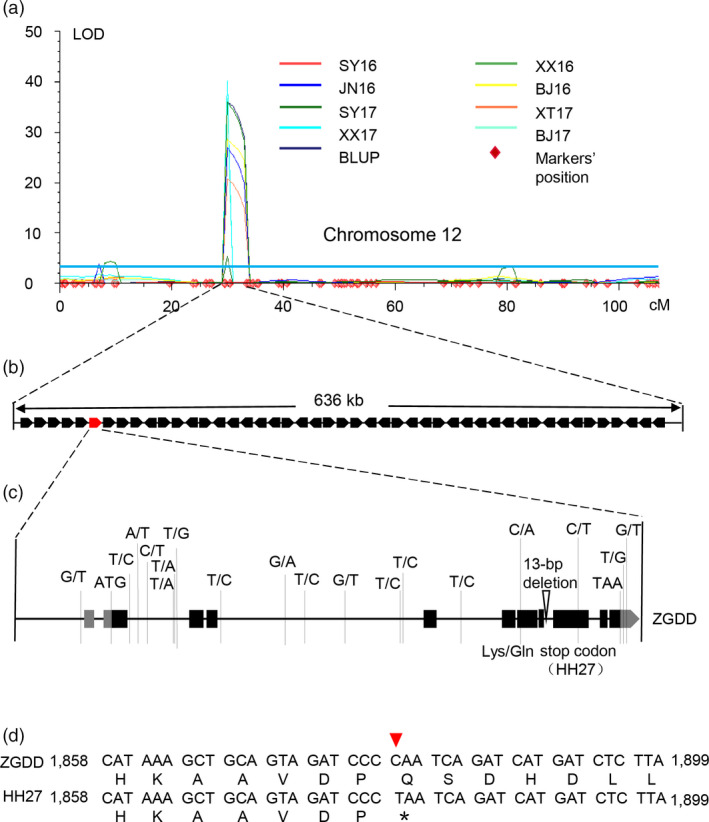
Mapping and subsequent cloning of the flowering time QTL *qFT12‐2*. (a) Chromosome 12 harbours a major QTL, *qFT12‐2*, identified via QTL mapping using the flowering time data for the ZGDD × HH27 RIL population grown under eight environments (SY17, XT17, XX16, XX17, JN16, BJ16 and BJ17) and using BLUP values. BLUP values of flowering time for each line were obtained across eight environments. (b) The 636‐kb genomic region between markers Chr12‐5445349 and Chr12‐6081748 in the Williams 82 reference genome contains 47 predicted genes. (c) Allelic variation in the candidate gene *Glyma.12G073900* between ZGDD and HH27. (d) Comparison of the *Glyma.12G073900* sequence in ZGDD and HH27. The arrowhead indicates the position of the non‐sense mutation in HH27. An asterisk indicates the termination of translation.

*qFT12‐2* was delimited to a 636‐kb region between markers Chr12‐5445349 and Chr12‐6081748 on chromosome 12, which harboured 47 genes according to the Williams 82 reference genome (Figure 1a, b, Table S6). One of these genes, *Glyma.12G073900* was homologous to *APRR7*, which was confirmed to participate in the circadian clock‐controlled flowering pathway in Arabidopsis (Nakamichi *et al.*, 2010; Nakamichi *et al.*, 2005). In addition, homologous genes in other crops have also been shown to regulate photoperiodic flowering (Beales *et al.*, 2007; Gao *et al.*, 2014; Koo *et al.*, 2013; Liu *et al.*, 2018a; Murphy *et al.*, 2011; Turner *et al.*, 2005; Yan *et al.*, 2013). Recently, a major QTL *qFT12‐1*, overlapping with *qFT12‐2* identified in our study, was mapped to a 56.4‐kb region, harbouring only four annotated genes, of which the Arabidopsis *PRR7* homologue *Glyma.12G073900* was confirmed to be the strongest candidate gene for *qFT12‐1* (Li *et al.*, 2019b). This suggested that *Glyma.12G073900* was the likely candidate gene underlying *qFT12‐2*.

### Sequence analysis of *Glyma.12G073900* in the RIL parents

We cloned and sequenced the genomic region of *Glyma.12G073900* in the two parents. Analysis of the 16 508 bp *Glyma.12G073900* sequence, which included 2554 bp of sequence upstream of the start codon, the 12 968 bp predicted coding region and 986 bp of the 3′ UTR, revealed 18 SNPs and one insertion/deletion polymorphism between ZGDD and HH27 (Figure 1c). One SNP in exon 6 results in variation at the 378th amino acid (glutamine in ZGDD and lysine in HH27). A non‐sense mutation (C1879T) in exon 8 of *Glyma.12G073900* was identified in HH27, resulting in premature termination of translation after 626 amino acids, whereas the Glyma.12G073900 protein of ZGDD is predicted to be 789 amino acids in length (Figure 1c, d). Moreover, this non‐sense mutation causes the loss of the CCT domain (Figure S3d), which functions in nuclear localization in CONSTANS family proteins (Robson *et al.*, 2001). We also genotyped the non‐sense variant (C1879T, Chr12‐5520945) in the RIL population using Kompetitive allele specific PCR (KASP). Among three markers (Chr12‐5502184, Chr12‐5520945 and Chr12‐6081748) within the *qFT12‐2* interval, Chr12‐5520945 was the most strongly associated with flowering time across all environments except SY16 (*P* < 0.01) (Figure S4). These findings support the inference that *Glyma.12G073900* is the candidate gene responsible for the *qFT12‐2* QTL.

In phylogenetic analysis, Glyma.12G073900 showed slightly higher amino acid sequence similarity with APRR3 than APRR7 (Figure S3a). The N‐terminal response‐regulator receiver domain and the C‐terminal CCT domain are conserved between the PRRs and ZGDD Glyma.12G073900 (Figure S3b,d). However, Glyma.12G073900 in ZGDD contains an EAR motif (LxLxL), which is required for the repressive activity of three APRR proteins (APRR9/7/5) and is not conserved in APRR3 (Figure S3c). Therefore, we hereafter refer to *Glyma.12G073900* as *GmPRR37*.

### *GmPRR37* displays a constitutive and diurnal expression pattern

**Figure 2:**
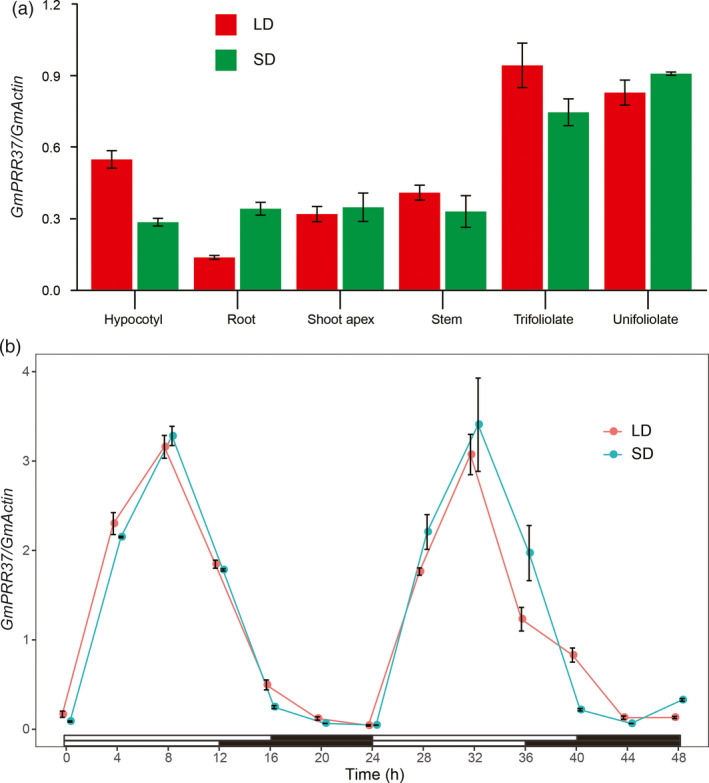
Expression pattern of *GmPRR37*. (a) Expression levels of *GmPRR37* in different organs of ZGDD plants on day 14 after commencing long‐day (LD, 16 h: 8 h, light: dark) or short‐day (SD, 12 h: 12 h, light: dark) treatment. Samples were collected 8 h after the lights were turned on. (b) Expression levels of *GmPRR37* in trifoliate leaves of ZGDD throughout a 48‐h period on days 10 and 11 of LD or SD treatment. The relative expression levels are normalized to *GmActin*. The data are means ± SE of three biological replicates.

The 2370 bp coding sequence (CDS) of *GmPRR37* was cloned from the late‐flowering cultivar ZGDD. We examined the expression levels of *GmPRR37* in different organs (root, hypocotyl, unifoliate leaf, trifoliolate leaf, stem and shoot apex) of SD‐ and LD‐treated ZGDD plants on day 14 of the photoperiod treatment. Under both LD and SD (LD, 16 h: 8 h, light: dark; SD, 12 h: 12 h, light: dark) conditions, *GmPRR37* was primarily expressed in unifoliate and trifoliolate leaves and had lower expression levels in the other tissues (Figure 2a). We also investigated the diurnal expression pattern of *GmPRR37* in trifoliolate leaves throughout the course of days 10 and 11 (13 time points). Under both LD and SD conditions, *GmPRR37* showed diurnal expression that peaked in the afternoon ~8 h after the lights were turned on (Figure 2b), indicating that *GmPRR37* expression is modulated by the circadian clock. A previous study revealed that the *OsPRR37* transcript exhibits a diurnal pattern in leaves with a maximum at Zeitgeber 8 under both SD and LD conditions (Gao *et al.*, 2014), indicating that *GmPRR37* may function similarly to *OsPRR37* in flowering regulation.

### CRISPR/Cas9‐induced *Gmprr37* mutants exhibit early flowering phenotype

**Figure 3:**
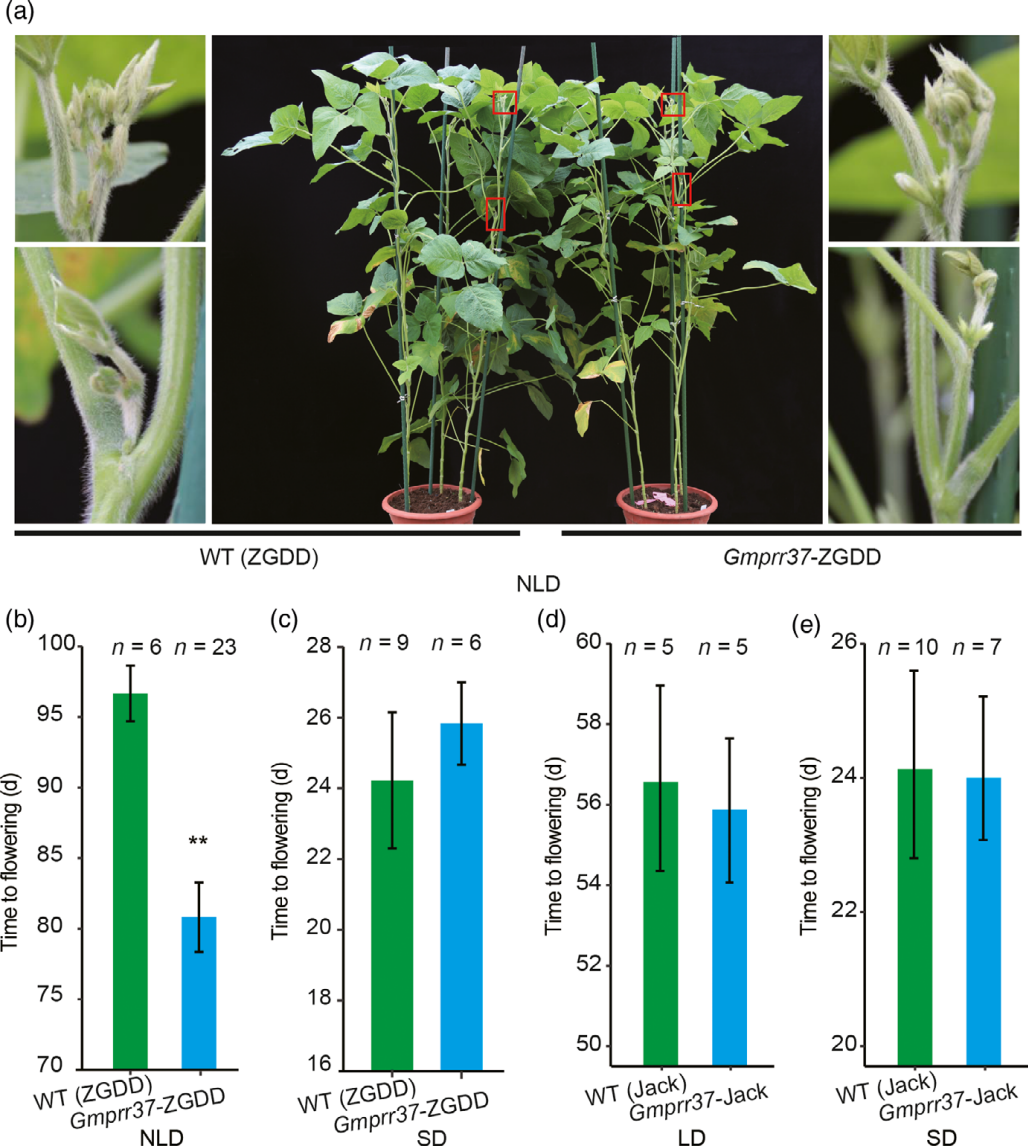
Phenotypes of the CRISPR/Cas9‐induced *Gmprr37* mutants. (a) Images of WT (cv ZGDD) and homozygous T2 *Gmprr37* mutants under natural long‐day conditions (NLD, 23rd June‐5th October) in Beijing, China (39°57′N, 116°19′E) (middle panel). A magnified view of the area in the red box is shown in the left and right panel. (b) Flowering time (days) of WT (cv ZGDD) plants and homozygous *Gmprr37*‐ZGDD mutants under NLD conditions. (c) Flowering time (days) of WT (cv ZGDD) plants and homozygous *Gmprr37*‐ZGDD mutants under SD (12 h: 12 h, light: dark) conditions. (d) Flowering time (days) of WT (cv Jack) plants and homozygous *Gmprr37*‐Jack mutants under LD (16 h: 8 h, light: dark) conditions. (e) Flowering time of WT (cv Jack) plants and homozygous *Gmprr37*‐Jack mutants under SD (12 h: 12 h, light: dark) conditions. The exact numbers of individual plants are shown. The flowering time is shown as the mean ± standard deviation, and statistical significance was determined using Student's *t* tests: **, *P* < 0.01.

To determine whether the mutation in the *GmPRR37* candidate gene is responsible for flowering time, we used the CRISPR/Cas9 system to generate targeted mutagenesis of the *GmPRR37* in soybean cultivar ZGDD and *Gmprr37* in Jack. Sequence analysis showed that Jack and HH27 had an identical non‐sense mutation resulting in premature termination of the predicted GmPRR37 protein compared with ZGDD. The target site (named *GmPRR37*‐CP) in the first exon of *GmPRR37* (ZGDD) and *Gmprr37* (Jack) was chosen (Figure S5a), and the mutants induced by CRISPR/Cas9 for the two cultivars were respectively named *Gmprr37*‐ZGDD (1‐bp deletion) and *Gmprr37*‐Jack (11‐bp deletion) (Figure S5b,c,d,e). In the T2 generation, homozygous *Gmprr37*‐ZGDD mutants induced by CRISPR/Cas9 displayed 15.8 days earlier in flowering time compared with WT (ZGDD) under NLD conditions (23rd June‐5th October, 2019) in Beijing, China (39°57′ N, 116°19′ E) (Figure 3a,b). However, the flowering times of the *Gmprr37*‐ZGDD mutants were basically the same as WT plants under SD (12 h: 12 h, light: dark) conditions: 25.8 ± 1.2 DAE for *Gmprr37*‐ZGDD mutants vs. 24.2 ± 1.9 DAE for WT (Figure 3c). We also examined the flowering time of WT (Jack) plants and *Gmprr37*‐Jack mutants under both SD and LD (SD, 12 h: 12 h, light: dark; LD, 16 h: 8 h, light: dark) conditions, the flowering time of *Gmprr37*‐Jack mutants were almost the same as WT plants: 24.1 ± 1.1 DAE for *Gmprr37*‐Jack mutants vs. 24.2 ± 1.4 DAE for WT under SD conditions and 55.8 ± 1.8 DAE for *Gmprr37*‐Jack mutants vs. 56.6 ± 2.3 DAE for WT under LD conditions (Figure 3d,e). These results verify that *GmPRR37* is the gene for the *qFT12‐2* QTL and suppresses flowering under LD conditions.

### Overexpression of *GmPRR37* in soybean delays flowering

**Figure 4:**
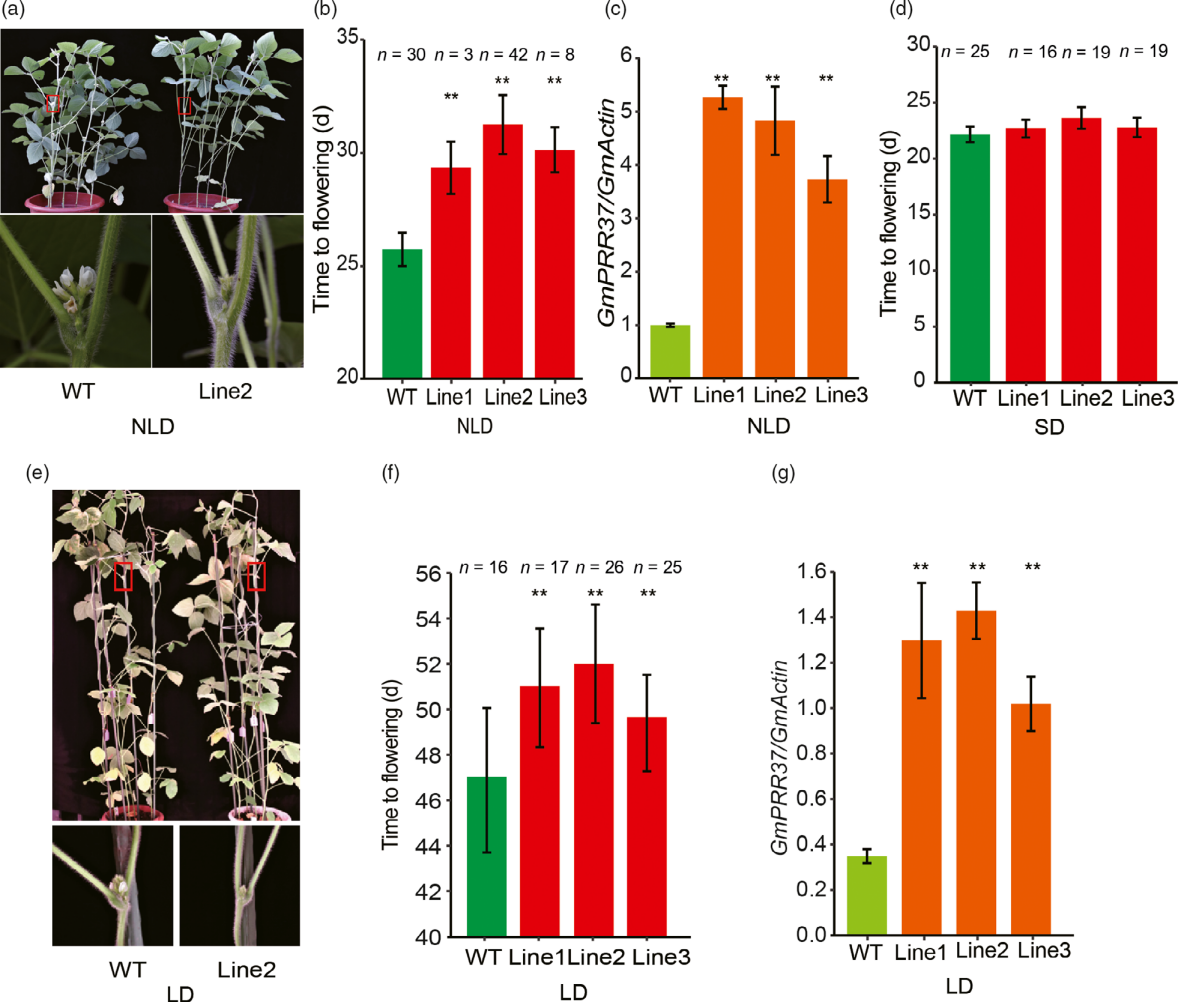
Phenotypes of the *GmPRR37* transgenic soybean plants. (a) Images of WT (cv Jack) and *GmPRR37* overexpression line 2 plants under natural long‐day conditions (NLD, 23rd July‐27th August) in Beijing, China (39°57′ N, 116°19′ E) (upper panel). A magnified view of the area in the red box is shown in the lower panel. (b) Flowering times of three *GmPRR37* overexpression lines and wild‐type (WT) plants under NLD conditions. The exact numbers of individual plants are shown. The flowering time is shown as the mean ± standard deviation, and statistical significance was determined using Student's *t* tests: **, *P* < 0.01. (c) Expression levels of *GmPRR37* in leaves at 25 DAE (day after emergence) under NLD conditions. Error bars indicate the SE values of three replications, and statistical significance was determined using Student's *t* tests: **, *P* < 0.01. (d) Flowering times of three *GmPRR37* overexpression lines and WT plants under SD (12 h: 12 h, light: dark) conditions. (e) Images of WT and *GmPRR37* overexpression Line 2 plants under LD (16 h: 8 h, light: dark) conditions (upper panel), and a close‐up view of the areas framed by the red boxes (lower panel). (f) Flowering time (days) of three *GmPRR37* overexpression lines and WT plants under LD conditions. Flowering time is shown as the mean ± standard deviation, and statistical significance was determined using Student's *t* tests: **, *P* < 0.01. (g) Expression levels of *GmPRR37* in leaves at 35 DAE under LD conditions. Error bars indicate the SE values of three replications, and statistical significance was determined using Student's *t* tests: **, *P* < 0.01.

For further verification of the role of *GmPRR37* in flowering time control, we generated a construct containing the CDS of ZGDD *GmPRR37* driven by the CaMV35S promoter and transformed it into the soybean cultivar Jack. Under NLD conditions (23rd July‐27th August) in Beijing, China (39°57′ N, 116°19′ E), the flowering times of three independent transgenic lines were significantly delayed by 3.6, 5.5 and 4.4 days compared with the recipient parent Jack (*P* < 0.01) (Figure 4a, b, c). Furthermore, under LD (16 h: 8 h, light: dark) conditions, the three transgenic lines flowered 4.1, 5.1 and 2.5 days later than the wild‐type (WT) plants (*P* < 0.01) (Figure 4e,f,g). However, the *GmPRR37* transgenic line 1 (22.7 ± 0.8 day after emergence, DAE), line 2 (23.6 ± 0.9 DAE) and line 3 (22.8 ± 0.9 DAE) flowered at the same time as WT (22.2 ± 0.7 DAE) under SD (12 h: 12 h, light: dark) conditions (Figure 4d). These results further confirm that *GmPRR37* is the correct *qFT12‐2* candidate gene and also acts as a flowering suppressor in soybean, especially under LD conditions.

### *GmPRR37* delays flowering time by promoting *GmFT1a* and suppressing *GmFT2a* and *GmFT5a* under LD conditions

**Figure 5:**
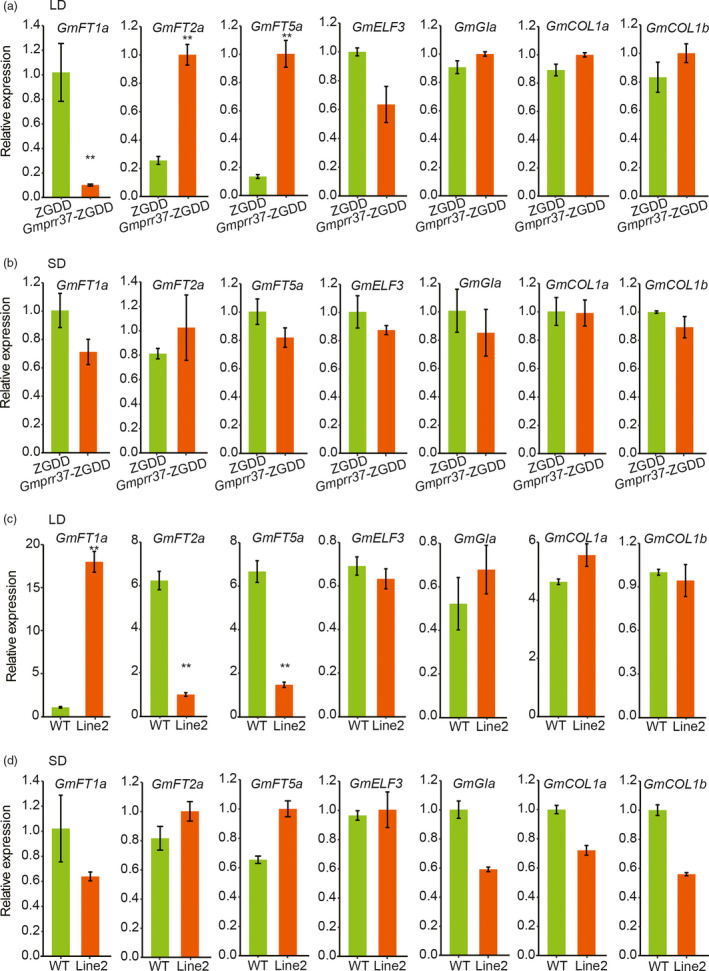
Expression levels of flowering‐related genes in leaves of the WT plants, CRISPR/Cas9‐induced *Gmprr37‐*ZGDD mutants and transgenic plants overexpressing *GmPRR37* under LD and SD conditions. (a) Expression analysis in the WT plants (cv ZGDD) and CRISPR/Cas9‐induced *Gmprr37*‐ZGDD mutants under LD (13.5 h: 10.5 h, light: dark) conditions. (b) Expression analysis in the WT plants (cv ZGDD) and CRISPR/Cas9‐induced *Gmprr37*‐ZGDD mutants under SD (12 h: 12 h, light: dark) conditions. (c) Expression analysis in the WT plants (cv Jack) and *GmPRR37* overexpression line 2 under LD (16 h: 8 h, light: dark) conditions. (d) Expression analysis in the WT plants (cv Jack) and *GmPRR37* overexpression line 2 under SD (12 h: 12 h, light: dark) conditions. Relative transcript levels of these genes were normalized to *GmActin*. Error bars indicate the SE values of three replications. Statistical significance was determined using Student's *t* tests: **, *P* < 0.01.

To investigate the relationship between *GmPRR37* and genes involved in photoperiodic flowering, we examined the expression of *GmFT1a*, *GmFT2a*, *GmFT5a*, *J* (*GmELF3*), *E2* (*GmGIa*), *GmCOL1a* and *GmCOL1b, *which are known to play key roles in photoperiod‐controlled flowering pathway, in CRISPR/Cas9‐induced *Gmprr37*‐ZGDD mutants, *Gmprr37*‐Jack mutants and transgenic plants overexpressing *GmPRR37* under LD and SD conditions. The results indicated that *GmFT2a* and *GmFT5a* expression were significantly up‐regulated and *GmFT1a* expression was down‐regulated in the *Gmprr37*‐ZGDD mutants under LD (13.5 h: 10.5 h, light: dark) conditions (Figure 5a). Meanwhile, the expression of *GmELF3*, *GmGIa*, *GmCOL1a* and *GmCOL1b* was not affected in the *Gmprr37*‐ZGDD mutants under LD conditions (Figure 5a). Whereas all the seven genes expression were not altered in the *Gmprr37*‐ZGDD mutants under SD (12 h: 12 h, light: dark) conditions (Figure 5b). As shown in Figure S6, the expression levels of these seven genes were not affected in the *Gmprr37*‐Jack mutants, findings consistent with no significant phenotypic alterations in flowering time observed under both SD and LD (SD, 12 h: 12 h, light: dark; LD, 16 h: 8 h, light: dark) conditions (Figure 3d, e).

Compared with WT plants, the expression of flowering‐inhibiting gene *GmFT1a* was significantly higher, and the flowering‐promoting gene *GmFT2a* and *GmFT5a* were significantly lower in the leaves of the *GmPRR37* overexpression plants under LD (16 h: 8 h, light: dark) conditions (Figure 5c). The expression levels of *GmELF3*, *GmGIa*, *GmCOL1a* and *GmCOL1b* were similar between overexpression line 2 and WT under LD conditions (Figure 5c). Under SD (12 h: 12 h, light: dark) conditions, the expression levels of these seven genes were not affected in the *GmPRR37* overexpression plants (Figure 5d). The results of these LD and SD experiments demonstrate that *GmPRR37* functions as a repressor of *GmFT2a* and *GmFT5a*, and an activator of *GmFT1a* to delay flowering in LD conditions.

### Geographical distribution of naturally occurring alleles and haplotypes

**Figure 6:**
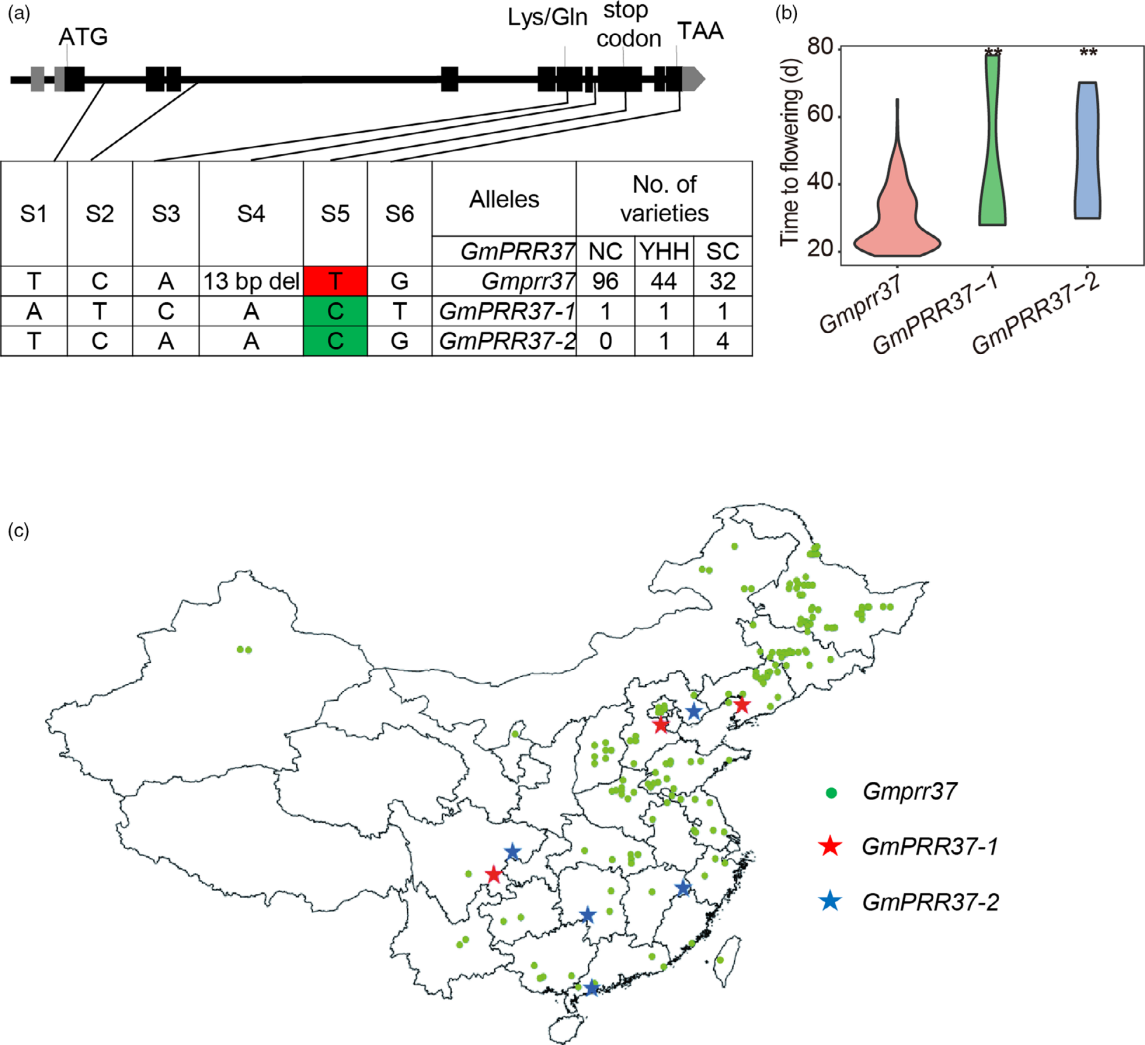
Identification of haplotypes in soybean cultivars. (a) Natural variation in nucleotide sequence of *GmPRR37* in soybean cultivars. (b) Flowering time (days) of cultivars with different *GmPRR37* alleles. Statistical significance was determined using Student's *t* tests: **, *P* < 0.01. (c) Geographical distribution of different haplotypes in China. BLUP values for flowering time for each cultivar were used for statistical analysis.

To evaluate the effect of mutations in *GmPRR37* on soybean adaptation, we determined the genotypes of 180 re‐sequenced soybean cultivars that have been conventionally cultivated in China (Table S7). We found five SNPs and one indel in the full‐length *Glyma.12g073900* sequence (Gm12:5508365‐5522772). SNPs S1 and S2, and indel S3 are intron variants, SNP S6 is a 3′ UTR variant, SNP S4 is a mis‐sense variant (Lys to Gln) and SNP S5 is a non‐sense variant (Figure 6a). Only two SNPs (S4 and S5) are located in the CDS. All six polymorphisms coincide with those identified between the RIL parents HH27 and ZGDD (Figure 1c). Based on the polymorphisms, we found one nonfunctional variant, *Gmprr37*, and two functional variants *GmPRR37‐1* and *GmPRR37‐2* (Figure 6a, Table S7). Among the 180 cultivars, *Gmprr37* was the most widely distributed and was significantly associated with earlier flowering time compared with *GmPRR37‐1* and *GmPRR37‐2* (Figure 6b). The allele frequency of the functional variants (*GmPRR37‐1* and *GmPRR37‐2*) declined from 13.51% in South China cultivars to 4.35% in Yellow‐Huai‐Hai Region cultivars and 1.03% in Northeast China cultivars. Furthermore, no cultivars harboured *GmPRR37* in the northern‐limit regions (from 40.45°N to 53°N) in China (Figure 6c). We have shown that overexpression of *GmPRR37* can significantly delay soybean flowering under long photoperiod conditions (Figure 4). Taken together, the effect of the functional variants on delayed flowering was gradually enhanced with the increase of day length from low to high latitudes; thus, germplasms with dominant alleles were artificially excluded during soybean cultivar improvement in higher latitudes in China. These findings indicate that natural variations in *GmPRR37* play an important role in soybean adaptation from low to high latitudes, and especially in the ability of soybean cultivars to grow in higher latitude regions with long photoperiods.

## Discussion

### *GmPRR37* is responsible for *qFT12‐2* in soybean

Flowering time is a critical determinant of the regional adaptation of soybean. Previous studies have uncovered a number of flowering‐time loci, which are catalogued in SoyBase (http://soybase.org/). In the current study, *qFT12‐2*, a major effect QTL controlling flowering time, was identified across different photoperiodic environments using 308 RILs derived from a cross between the late‐flowering soybean cultivar ZGDD and the early‐flowering soybean cultivar HH27 (Figure 1a). Our comparative analysis showed that *qFT12‐2* overlapped with the recently reported QTL *qFT12.1* uncovered by linkage analysis (Liu *et al.*, 2018b) and the locus *SAL10* identified in a GWAS study (Fang *et al.*, 2017). Bioinformatics analysis revealed that *GmPRR37*, a homologue of *PRRs*, which have been demonstrated to act as regulators of photoperiod flowering in various plant species (Beales *et al.*, 2007; Gao *et al.*, 2014; Koo *et al.*, 2013; Liu *et al.*, 2018a; Murphy *et al.*, 2011; Nakamichi *et al.*, 2010; Turner *et al.*, 2005; Yan *et al.*, 2013), was a strong candidate gene for *qFT12‐2* (Figure 1, Figure S3, Table S6). *GmPRR37* from the RIL parent HH27 carries a non‐sense mutation that leads to the deletion of the CCT domain in the encoded protein (Figure S3d). Functional *GmPRR37* alleles were strongly associated with late‐flowering time in conventionally cultivated germplasms (Figure 6b). To further investigate the function of *GmPRR37*, genetic transformation was conducted in soybean. We found that overexpression of *GmPRR37* in soybean significantly delayed flowering time, and CRISPR/Cas9‐induced *Gmprr37* mutants exhibited early flowering under long photoperiod conditions (Figures 3 and 4). The results from linkage, bioinformatics and transgenic transformation analysis support the conclusion that *GmPRR37* is the causal gene of *qFT12‐2*.

### *GmPRR37* acts as a suppressor in an LD‐dependent flowering pathway

Overexpression of *OsPRR37* in rice significantly delays flowering time (Liu *et al.*, 2018a), and *Osprr37* mutants show reduced photoperiod sensitivity and flower earlier than WT (Koo *et al.*, 2013). However, in Arabidopsis, mutants of *APRRs* are associated with late flowering (Murakami *et al.*, 2004; Nakamichi *et al.*, 2005). These findings suggest that there is functional differentiation of *PRRs* between LD and SD plants.

In this study, CRISPR/Cas9‐induced *Gmprr37*‐ZGDD mutants showed significantly early flowering under NLD conditions, but the flowering time was almost the same between *Gmprr37*‐ZGDD mutants and WT plants under SD conditions (Figure 3). Transgenic overexpression of *GmPRR37* significantly delayed flowering under long photoperiod conditions, while the *GmPRR37* overexpression lines showed no alteration in flowering time under SD conditions (Figure 4). These results suggest that *GmPRR37* acts as a suppressor in LD‐dependent flowering pathway in soybean. Under LD conditions, *GmPRR37* down‐regulates the expression of the flowering‐promoting *FT* homologues *GmFT2a* and *GmFT5a* (Figure 5). Similarly, *OsPRR37* acts as a LD‐specific suppressor of rice florigen (*Hd3a*) and a rice‐specific flowering integrator (*Ehd1*) (Gao *et al.*, 2014; Yan *et al.*, 2013), and its activity is gradually enhanced with increased day length, causing a more significant delay in rice flowering (Gao *et al.*, 2014). Furthermore, *GmPRR37* exhibits the same diurnal expression pattern as *OsPRR37* in rice, which is regulated by the circadian clock under both LD and SD conditions (Gao *et al.*, 2014). Whereas, *GmPRR37* up‐regulates the expression of the flowering‐inhibiting *FT* homologue *GmFT1a*, which has opposite roles of flowering‐promoting genes *GmFT2a/5a* (Liu *et al.*, 2018c). This finding demonstrates that *GmPRR37* confers an expanded regulation mechanism of circadian clock on photoperiodic flowering pathways in soybean. Our insights into *GmPRR37* will facilitate the investigation and characterization of the circadian clock and photoperiodic flowering in soybean.

### *GmPRR37* provides an opportunity to improve soybean adaptation to diverse geographic regions and farming systems

Soybean is cultivated worldwide in a broad range of latitudes, although each cultivar is restricted to a relatively narrow latitude range. This wide distribution of soybean species results from rich natural variations and different combinations of genes and QTLs controlling flowering time. For example, natural mutants of *GmELF3* (Yue *et al.*, 2017) and *ft2aft5a* double mutants created by CRISPR/Cas9 (Cai *et al.*, 2020) can improve soybean adaptation to tropical and low‐altitude regions, whereas natural variations in *GmGBP1* are associated with soybean ecological adaptation to high latitudes (Zhao *et al.*, 2018). Various combinations of mutations at the *E* loci (*E1*, *E2*, *E3* and *E4*) provided considerable genetic plasticity that contributed to soybean cultivation at diverse latitudes (Jiang *et al.*, 2014; Tsubokura *et al.*, 2014).

In the current study, we found that one nonfunctional variant *Gmprr37*, which is significantly associated with early‐flowering time, has contributed to soybean adaptation to higher latitude regions (Figure 6). CRISPR/Cas9‐mediated targeted mutagenesis of *Gmprr37* mutants in a very late‐flowering soybean cultivar ZGDD displayed early flowering by about 15.8 days under NLD conditions (Figure 3). It is conceivable that the CRISPR/Cas9 system can be employed to knockout *GmPRR37* in late‐flowering soybean cultivars and thus enable these cultivars to be introduced to higher latitudes or planted in spring in multiple cropping systems. Transgenic overexpression of *GmPRR37* significantly delayed flowering and maturation when sown in summer of Beijing (Figure 4); thus, *GmPRR37* could be used to breed cultivars with longer growth period in temperate region. Furthermore, flowering was significantly delayed in *GmPRR37* backgrounds with various *E* loci genotypes compared with *Gmprr37* (Figure S6). Our identification of *GmPRR37* may provide opportunities to breed highly optimized cultivars with flexible adaptation to specific regions and farming systems.

We also investigated whether flowering time variations could be attributed to haplotype combinations of *GmPRR37*, *E1*, *E2*, *FT5a*, *FT2a* and *E3* (genes underlying *qFT12‐2*, *qFT6*, *qFT10*, *qFT16‐1*, *qFT16‐2* and *qFT19*, respectively) in the RIL population and found that haplotype combinations of these loci could explain 63.73%, 81.69%, 76.41%, 82.13%, 33.59%, 73.55%, 74.49% and 81.16% of the flowering time variations in SY16, XX16, JN16, BJ16, SY17, XT17, XX17 and BJ17, respectively (Table S5). Notably, allelic combinations of these loci can explain a much larger percentage of variation in flowering time under the six environments with relatively longer daylengths (BJ16, BJ17, XX16, XX17, JN16 and XT17) but explain a substantially lower percentage of variance in the other two environments with SD conditions (SY16 and SY17). These observations indicate that selection of specific haplotype combinations of these genes could improve the regional fitness of soybean, especially under LD conditions. Further efforts to comprehensively analyse the respective contribution and allelic combinations of *GmPRR37*, classical *E* genes and other genetic loci controlling flowering time would facilitate the rational design of soybean cultivars with optimum adaptation to target photoperiod environments.

In summary, through map‐based cloning, genetic transformation and diversity analyses, we find that *GmPRR37* acts as a floral repressor under long photoperiod conditions and has contributed to soybean ecological adaption. Phylogenetic analysis showed that there are 14 soybean orthologs of Arabidopsis *PRR* genes. The expression patterns of *GmPRR37* showed a clear circadian rhythm. Therefore, we speculate that *GmPRRs* might play an important role in the circadian clock and photoperiod flowering in soybean. The findings of this study can facilitate the breeding of soybean adapted to diverse geographic regions and multiple farming systems.

## Experimental procedures

### RIL population construction and phenotypic analysis

A F_6:7_ recombinant inbred line (RIL) population derived from a cross between the very late‐flowering soybean cultivar ZGDD belonging to maturity group (MG) VIII and the extremely early‐flowering soybean cv HH27 (MG 0) were created using the single‐seed descent method. The cross was developed under SD conditions, followed by successive self‐pollination until the F_6_ generation in Sanya (Hainan province, China) (18°18′N, 109°30′E), which has short photoperiods. A set of 308 RILs along with the parents were planted during 2016 and 2017 at five locations in Beijing (40°13′N, 116°33′E) on July 3, 2016 and June 17, 2017, in Xinxiang (Henan province, China) (35°08′N, 113°45′E) on July 5, 2016 and June 22, 2017, in Sanya (Hainan province, China) (18°18′N, 109°30′E) on December 19, 2016 and March 18, 2017, in Jining (Shandong province, China) (35°27′N, 116°34′E) on July 3, 2016 and in Xiangtan (Hunan province, China) (27°40′N, 112°39′E) on June 20, 2017. Each location and year combination was considered as an experimental environment, and these eight environments were named BJ16, BJ17, XX16, XX17, SY16, SY17, JN16 and XT17, respectively. The RILs and parents were planted in a 1.5 m row, with 0.5 m separating rows and a space of 0.1 m between adjacent plants. All lines were arranged in a randomized complete block design with two replications. The flowering time of five plants was investigated in each replication. Flowering time was calculated as the period from emergence (VE) to beginning bloom (R1) as previously described (Fehr *et al.*, 1971).

The flowering‐time phenotype in a single environment was determined by taking the average for each family from two replications. To eliminate the influence of environmental effects on phenotypic variation, best linear unbiased predictor (BLUP) values of flowering time for each line were obtained across eight environments using a mixed linear model with the fitting of both genotype and environment as random effects using the R package ‘lme4’ (R Core Team, 2013).

### DNA extraction and genotyping

Genomic DNA was extracted and purified from the newly expanded trifoliate leaves of each of the parents and the 308 RILs using the Plant Genomic DNA Kit (Tiangen Biotech, Beijing, China). The 2b‐RAD libraries were prepared for the 310 samples as previously described (Wang *et al.*, 2012). Raw reads were trimmed to remove adaptor sequences, and the 3′ terminal positions of each read were eliminated. Reads with no restriction sites or containing ambiguous bases (N), low‐quality positions (>20% nucleotides with a Phred quality score <20), or long homopolymer regions (>8%) were removed. High‐quality reads were aligned using the SOAP software v2.21 (Li *et al.*, 2009). A maximum of two mismatches (–v 2) were allowed for each read, and those mapped onto more than one position in the genomic reference sequence were excluded (–r 0). The match mode was set to ‘find the best hits’ (–M 4). The SNPs were filtered with the RADtyping program v1.0 (Fu *et al.*, 2013) using the following criteria: (1) Polymorphic loci with more than two alleles were deleted; (2) segregating markers that could be genotyped in at least 80% of the individuals were kept for analysis; (3) all SNPs with a minor allele frequency (MAF) < 0.05 were removed; and (4) only one bi‐allelic SNP at each locus was retained.

### Genetic map construction and QTL analysis

Based on the genotypic data for 308 RILs, a genetic map with 20 linkage groups was constructed using the Kosambi mapping function of the Joinmap v4.1 software (Stam, 1993). The LOD threshold was set as 5.0 to determine the genetic position of each marker.

The additive QTLs for flowering time were detected using inclusive composite interval mapping of additive functionality (ICIM‐ADD) in the QTL IciMapping software v4.1 (Li *et al.*, 2008; Meng *et al.*, 2015). Missing phenotypes were deleted, and a 1 cM walk speed with a stepwise regression probability of 0.001 was used for QTL detection. The LOD value threshold for evaluating the significant QTLs was determined using 1,000 permutations at the 0.05 significance level. Thus, a LOD score of 3.29 was set to define the presence of a QTL. QTL designations were defined adopting the previously reported nomenclature (McCouch *et al.*, 1997).

### Analysis of diurnal expression patterns

For analysis of the expression pattern of *GmPRR37*, the soybean cultivar ZGDD was grown under SD (12 h: 12 h, light: dark) and LD (16 h: 8 h, light: dark) photoperiods. After entraining for 14 days, different organs (root, hypocotyl, unifoliate leaf, stem, trifoliolate leaf and shoot apex) were sampled in bulk from three plants for each treatment and then stored in liquid nitrogen. We also collected trifoliolate leaves at 4‐h intervals for a total of 48 h on days 10 and 11 of LD or SD treatment..

### RNA extraction, reverse transcription and quantitative real‐time PCR

For reverse transcription and quantitative real‐time PCR, total RNA was isolated from different tissues using the Trizol Up Plus RNA Kit (Tiangen Biotech). cDNAs were synthesized with Superscript II reverse transcriptase (TransGen Biotech). The qRT‐PCR was performed using the ABI7900 system (Applied Biosystems). The PCR reactions contained 1 μL of 1:5 diluted cDNA, 0.2 μL of gene‐specific primers, 0.2 μL of 50× ROX High Reference Dye, 5 μL of 2 ×KAPA SYBR^®^ FAST qPCR Master Mix (KAPA Biosystems) and water to a final volume of 10 μL. The PCR cycling conditions were as follows: 95 °C for 30 min followed by 40 cycles of 95 °C for 5 s and 60 °C for 30 s. Three biological replicates were performed for each reaction, and the qRT‐PCR data were analysed using SDS2.3 software. The primers used for real‐time quantitative PCR are listed in Table S8.

### Transformation of CRISPR/Cas9 in soybean

The CRISPR/Cas9 system was employed to knockout soybean *GmPRR37* and *Gmprr37*. The target site (referred to here as *GmPRR37*‐CP) in the first exon of *GmPRR37* and *Gmprr37* was selected (Figure S5a) using the web tool CRISPR‐P V2.0 (Liu *et al.*, 2017). Pairs of DNA oligonucleotides of the sgRNAs were synthesized by TSINGKE and annealed to generate dimers, which were subsequently integrated into the CRISPR/Cas9 expression vector as previously reported (Cai *et al.*, 2018). The vector was then transformed into *Agrobacterium tumefaciens* strain EHA105 via electroporation. The soybean cultivar ZGDD (*GmPRR37*) and Jack (*Gmprr37*) were used for transformation according to the previously reported protocol (Chen *et al.*, 2018). DNA extracted from leaf tissue was used to examine CRISPR/Cas9‐induced mutations at the target sites using PCR with the forward primer (5′‐GCGCTGACTTGACTGATGGATA‐3′) and reverse primer (5′‐ACAACATGGCGTGTCGAATC‐3′) and DNA sequencing analysis. Wild‐type ZGDD and its homozygous *Gmprr37* mutants induced by CRISPR/Cas9 were sown under NLD conditions in Beijing, China (39°57′ N, 116°19′ E) on 23 June 2019 and under SD (12 h: 12 h, light: dark) conditions. Wild‐type Jack and the homozygous *Gmprr37* mutants were grown under SD (12 h: 12 h, light: dark) and LD (16 h: 8 h, light: dark) photoperiodic conditions. The flowering time of each plant was recorded as days from VE to R1 as previously described (Fehr *et al.*, 1971). Data are shown as mean values ± one standard deviation, and Student's *t* tests were used to assess the significance of differences between lines.

### Transgenic overexpression in soybean

For construction of the overexpression vector, the coding region of *GmPRR37* from ZGDD, the *Hind*III and *Spe*I restriction sites and an additional 15 bp of the *Sp1300‐GFP* vector adjacent to restriction sites were PCR‐amplified. The primers are listed in Table S8. *GmPRR37* was cloned into the *Hind*III‐*Spe*I sites of the *Sp1300‐GFP* vector using the pEASY‐Uni Seamless Cloning and Assembly Kit (TransGen Biotech). From the resultant vector, an *Xba*I‐*Sal*I fragment containing the *GmPRR37::GFP* cassette was isolated and then inserted into the plant binary vector *pTF101.1*. *GmPRR37::GFP* expression was driven by a 2× CaMV 35S promoter. The final plasmid was introduced into *Agrobacterium tumefaciens* strain EHA101 and ultimately transformed into soybean cv ‘Jack’ following the cotyledon‐node method (Chen *et al.*, 2018). Transgenic plants were identified by daubing leaves with 160 mg/L glufosinate and detecting PAT proteins using Liberty Link strips. Putative transgenics were then subjected to molecular and phenotypic analysis. All transgenic and WT Jack plants were grown under SD (12 h: 12 h, light: dark), LD (16 h: 8 h, light: dark) and NLD (23rd July‐ 27th August, 2018, Beijing) conditions. The flowering time of each plant was recorded as days from VE to R1 as previously described (Fehr *et al.*, 1971). Data are shown as mean values ± one standard deviation, and Student's *t* tests were used to assess the significance of differences between lines.

### Haplotype analysis of *GmPRR37*

A diverse panel of 180 soybean cultivars, including 97 cultivars from Northeast China, 46 cultivars from Yellow‐Huai‐Hai, 37 cultivars from South China (Table S7), were re‐sequenced to investigate the natural variation of *GmPRR37*. These cultivars were chosen because they originated from different ecological regions and had extensive variation in flowering time.

The field trials with the panel of 180 soybean cultivars were performed during 2016 and 2017 at four locations: Beijing in 2016 and 2017, Xinxiang in 2016 and 2017, Jining in 2016 and Xiangtan in 2017, using a randomized complete block design with two replications. Each location and year combination was considered as an experimental environment. Flowering time was calculated as the period from VE to R1 (Fehr *et al.*, 1971). The BLUP value of flowering time for each cultivar was obtained across the six environments and used for further statistical analysis.

## Conflict of interest

The authors declare that they have no conflicts of interest.

## Author contributions

L.W. performed the experiments and wrote the manuscript. S.S. constructed the RIL population. T.W. and B.J. provided the data for the diverse panel of 180 soybean cultivars. L.L., X.S., J.L. and H.J. performed the phenotypic characterization. Y.C. assisted in soybean transformation. C.W., S.Y. and L.C. revised the manuscript. T.H. and W.H. conceived the research and revised the manuscript.

## Supporting information


**Figure S1** Frequency distribution of flowering time among 308 RILs in different environments and the distribution of the best linear unbiased predictors (BLUP).
**Figure S2 **High‐density genetic linkage map for soybean.
**Figure S3 **Phylogenetic analysis of GmPRR37.
**Figure S4 **Genetic loci associated with flowering time within the *qFT12‐2* interval.
**Figure S5 **Homozygous targeted mutagenesis of GmPRR37 (ZGDD) and *Gmprr37* (Jack) induced by CRISPR/Cas9.
**Figure S6 **Expression levels of flowering‐related genes in leaves of the WT plants (cv Jack) and CRISPR/Cas9‐induced *Gmprr37*‐Jack mutants under LD and SD conditions.
**Table S1 **Flowering times of RILs and parents grown under eight different environments and best linear unbiased predictor (BLUP).
**Table S2 **Statistics of 2b‐RAD reads and mapping rates of the RILs and parents (sequencing of DNA digested with *Bsa*XI).
**Table S3 **Statistics of 2b‐RAD reads and mapping rates of the RILs and parents (sequencing of DNA digested with *Fal*I).
**Table S4 **Description of characteristics of the 20 linkage groups in the high‐density genetic map.
**Table S5 **Putative QTLs for soybean flowering time identified using an RIL population grown in eight different environments and using BLUP values.
**Table S6 **Predicted genes located in the mapped 636‐kb genomic region of *qFT12‐2* in the Williams 82 reference genome.
**Table S7 **Haplotypes of *GmPRR37* in 180 soybean cultivars from China.
**Table S8 **Primer sequences used in this study.
