# Supplementary material for: Natural variation and CRISPR/Cas9‐mediated mutation in GmPRR37 affect photoperiodic flowering and contribute to regional adaptation of soybean
Source: Plant Biotechnol J. 2020 Feb 13;18(9):1869–81. doi: 10.1111/pbi.13346 (PMC7415786; doi:10.1111/pbi.13346)
Supplement: Supplementary file 1 — Figure S1 Frequency distribution of flowering time among 308 RILs in different environments and the distribution of the best linear unbiased predictors (BLUP). Figure S2 High‐density genetic linkage map for soybean. Figure S3 Phylogenetic analysis of GmPRR37. Figure S4 Genetic loci associated with flowering time within the qFT12‐2 interval. Figure S5 Homozygous targeted mutagenesis of GmPRR37 (ZGDD) and Gmprr37 (Jack) induced by CRISPR/Cas9. Figure S6 Expression levels of flowering‐related genes in leaves of the WT plants (cv Jack) and CRISPR/Cas9‐induced Gmprr37‐Jack mutants under LD and SD conditions. Table S1 Flowering times of RILs and parents grown under eight different environments and best linear unbiased predictor (BLUP). Table S2 Statistics of 2b‐RAD reads and mapping rates of the RILs and parents (sequencing of DNA digested with BsaXI). Table S3 Statistics of 2b‐RAD reads and mapping rates of the RILs and parents (sequencing of DNA digested with FalI). Table S4 Description of characteristics of the 20 linkage groups in the high‐density genetic map. Table S5 Putative QTLs for soybean flowering time identified using an RIL population grown in eight different environments and using BLUP values. Table S6 Predicted genes located in the mapped 636‐kb genomic region of qFT12‐2 in the Williams 82 reference genome. Table S7 Haplotypes of GmPRR37 in 180 soybean cultivars from China. Table S8 Primer sequences used in this study. [file PBI-18-1869-s001.pdf]

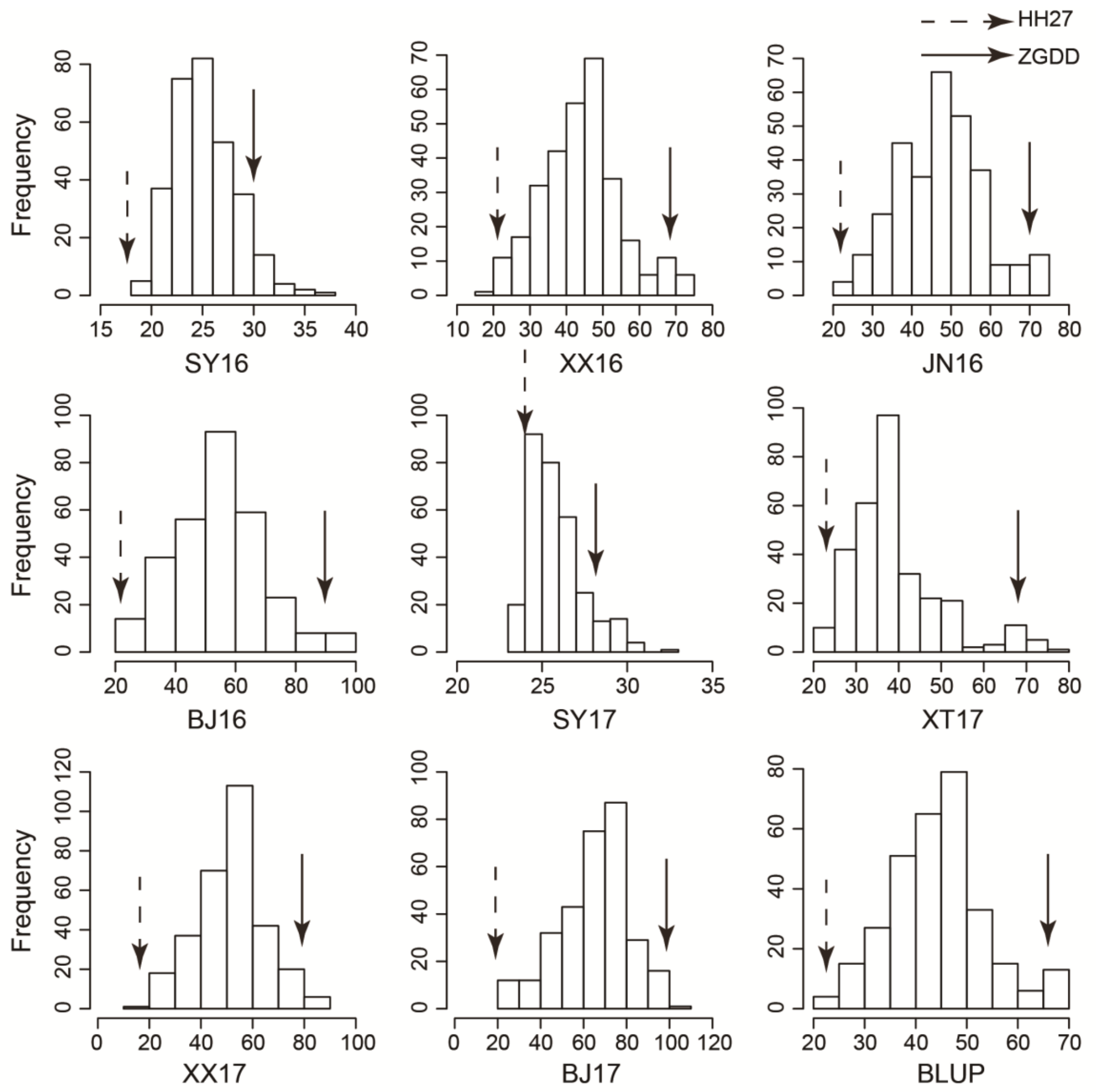

Figure S1 Frequency distribution of flowering time among 308 RILs in different environments and the distribution of the best linear unbiased predictors (BLUP). SY16, XX16, JN16 and BJ16 represent Sanya, Xinxiang, Jining and Beijing in 2016, respectively; SY17, XT17, XX17 and BJ17 represent Sanya, Xiangtan, Xinxiang and Beijing in 2017, respectively. BLUP values of flowering time for each line were obtained across eight environments. The arrows indicate the values of the parental lines.

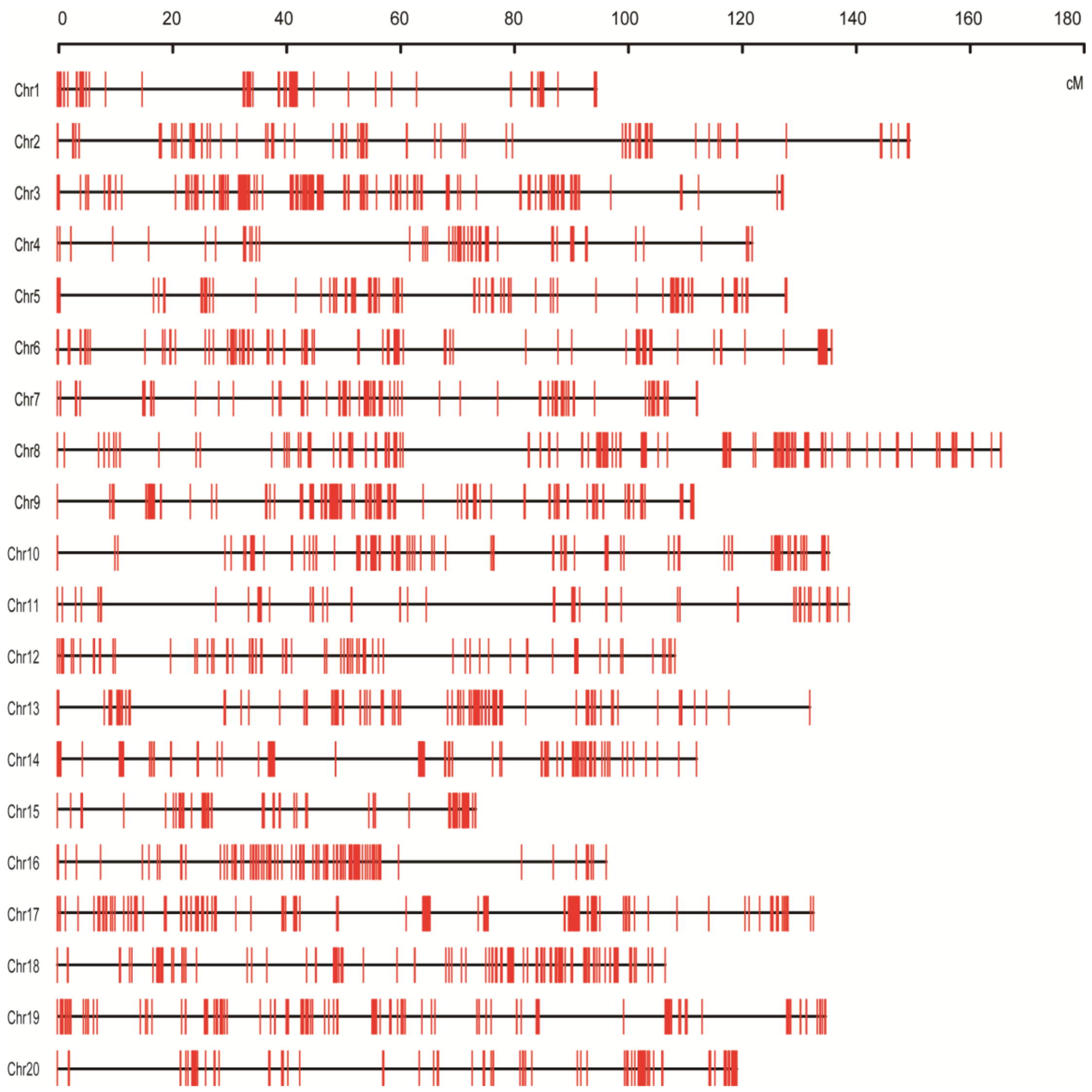

Figure S2 High-density genetic linkage map for soybean. A red bar means a SNP marker.

(a)

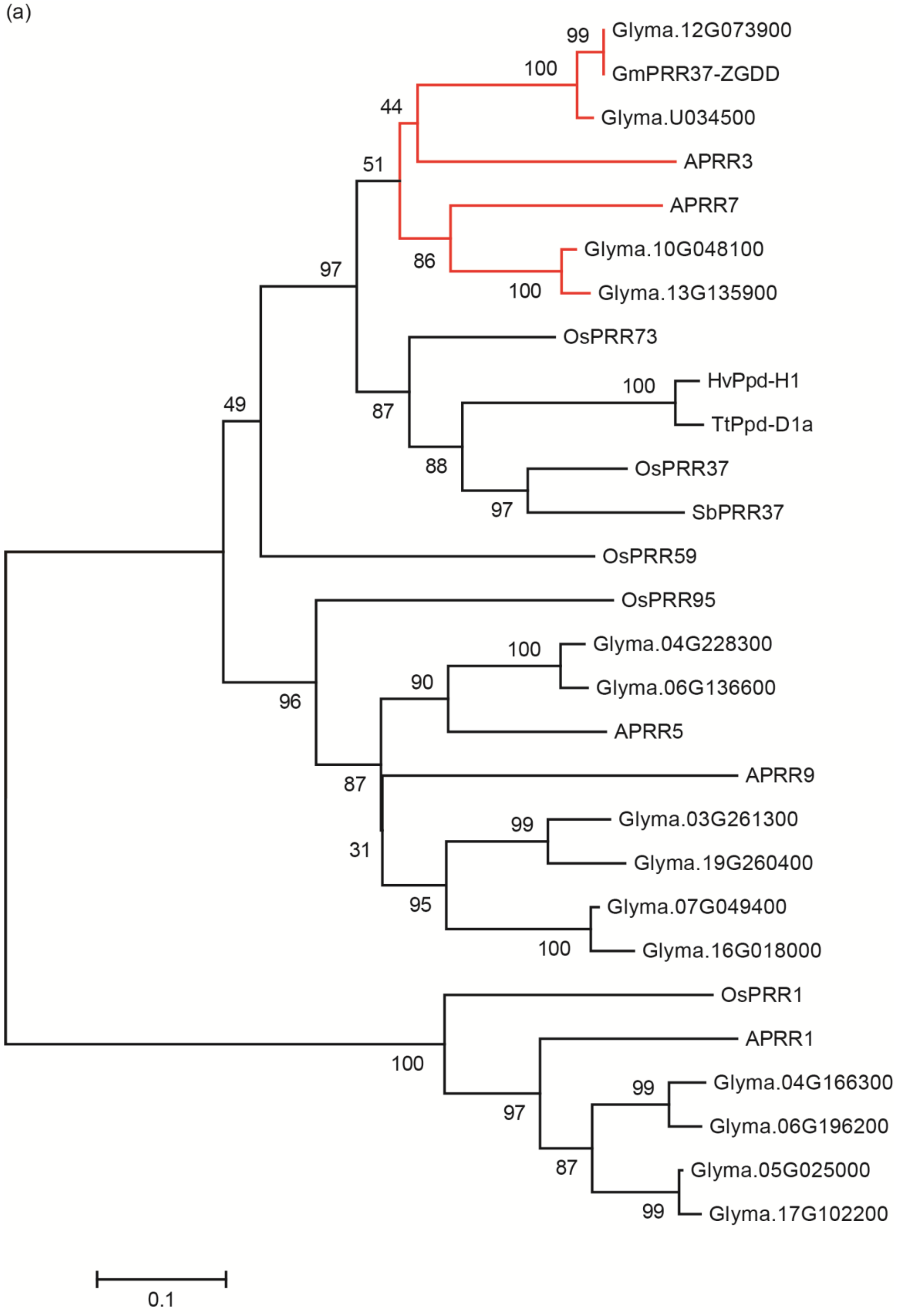
Glyma.12G073900
GmPRR37-ZGDD
Glyma.U034500
APRR3
APRR7
Glyma.10G048100
Glyma.13G135900
OsPRR73
HvPpd-H1
TtPpd-D1a
OsPRR37
SbPRR37
OsPRR59
OsPRR95
Glyma.04G228300
Glyma.06G136600
APRR5
APRR9
Glyma.03G261300
Glyma.19G260400
Glyma.07G049400
Glyma.16G018000
OsPRR1
APRR1
Glyma.04G166300
Glyma.06G196200
Glyma.05G025000
Glyma.17G102200
99
100
44
51
86
100
97
87
100
88
97
49
100
90
96
87
99
31
95
100
100
97
99
87
99
0.1

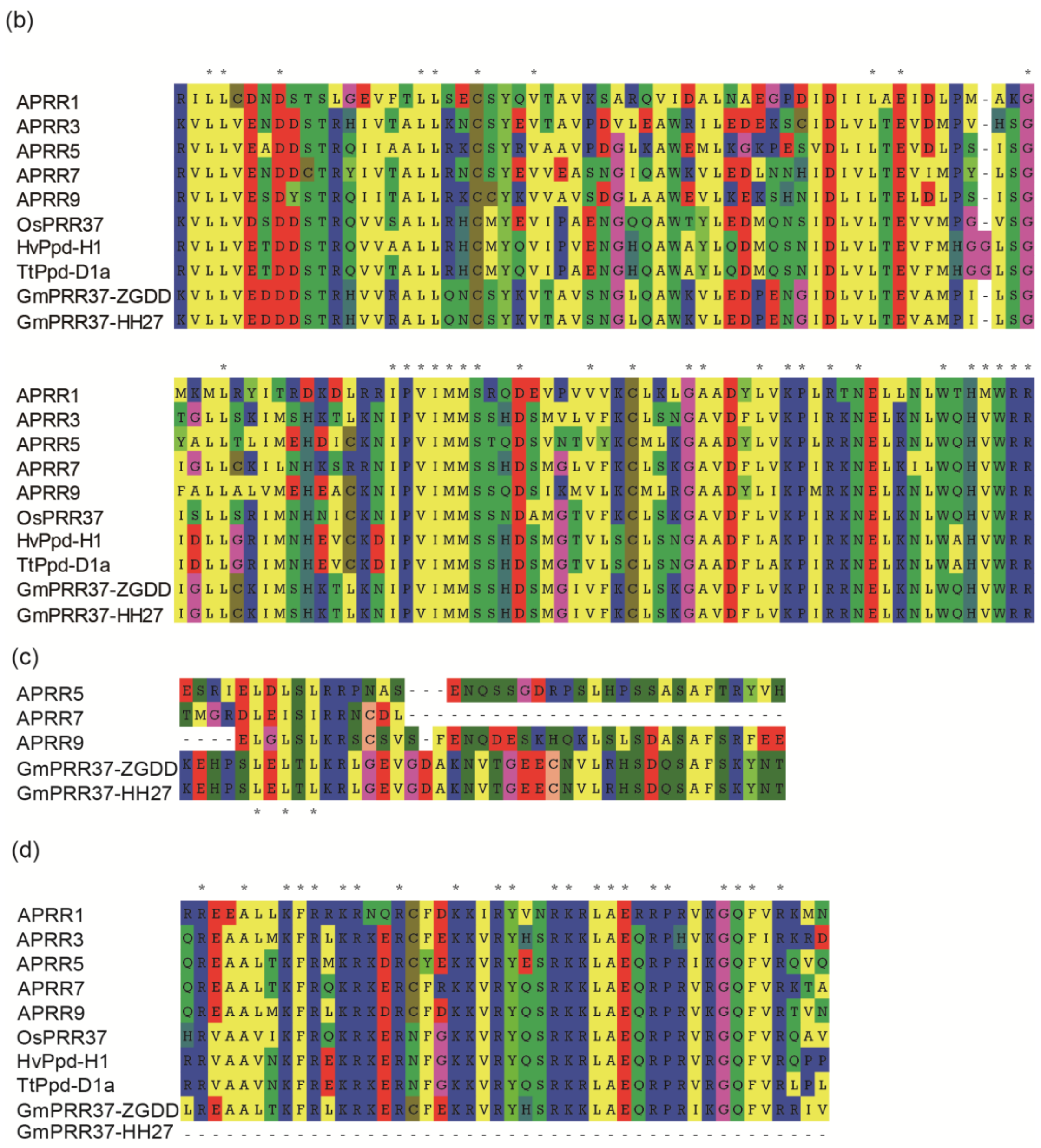

Figure S3 Phylogenetic analysis of GmPRR37. (a) Phylogenetic tree of PRR proteins in higher plants. Amino acid alignment of pseudo-receiver domains (b), EAR motifs (c), and CCT domains (d) of PRRs in higher plants. Asterisks indicate the amino acid residues conserved among PRRs.

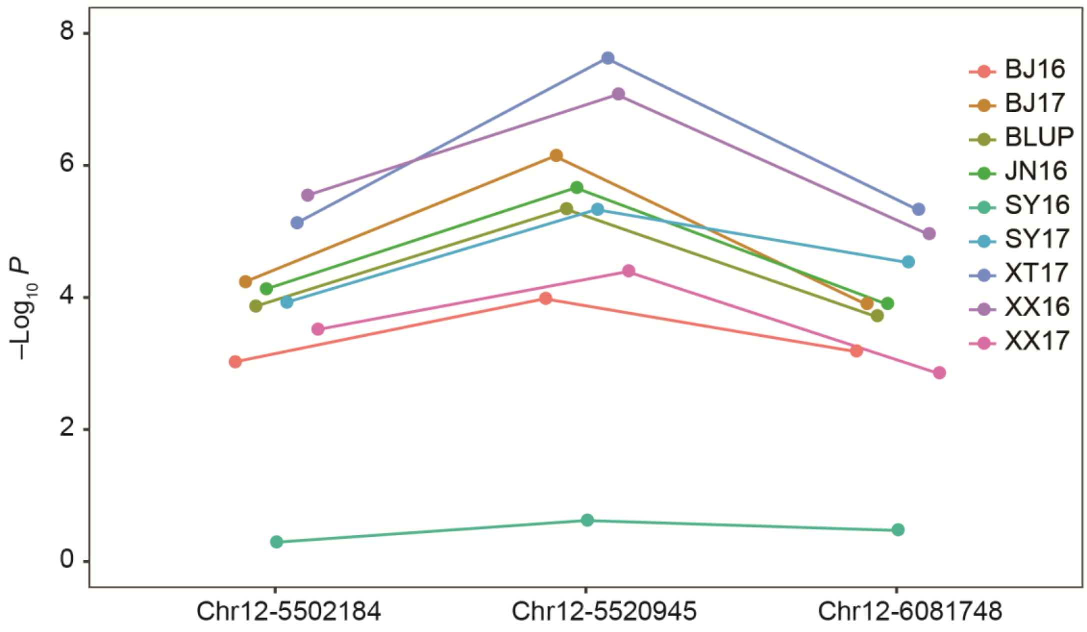

Figure S4 Genetic loci associated with flowering time within the *qFT12-2* interval. *P* values resulting from t-test of two genotypic classes in the RIL population. SY16, XX16, JN16 and BJ16 represent Sanya, Xinxiang, Jining and Beijing in 2016, respectively; SY17, XT17, XX17 and BJ17 represent Sanya, Xiangtan, Xinxiang and Beijing in 2017, respectively. BLUP represents best linear unbiased predictor.

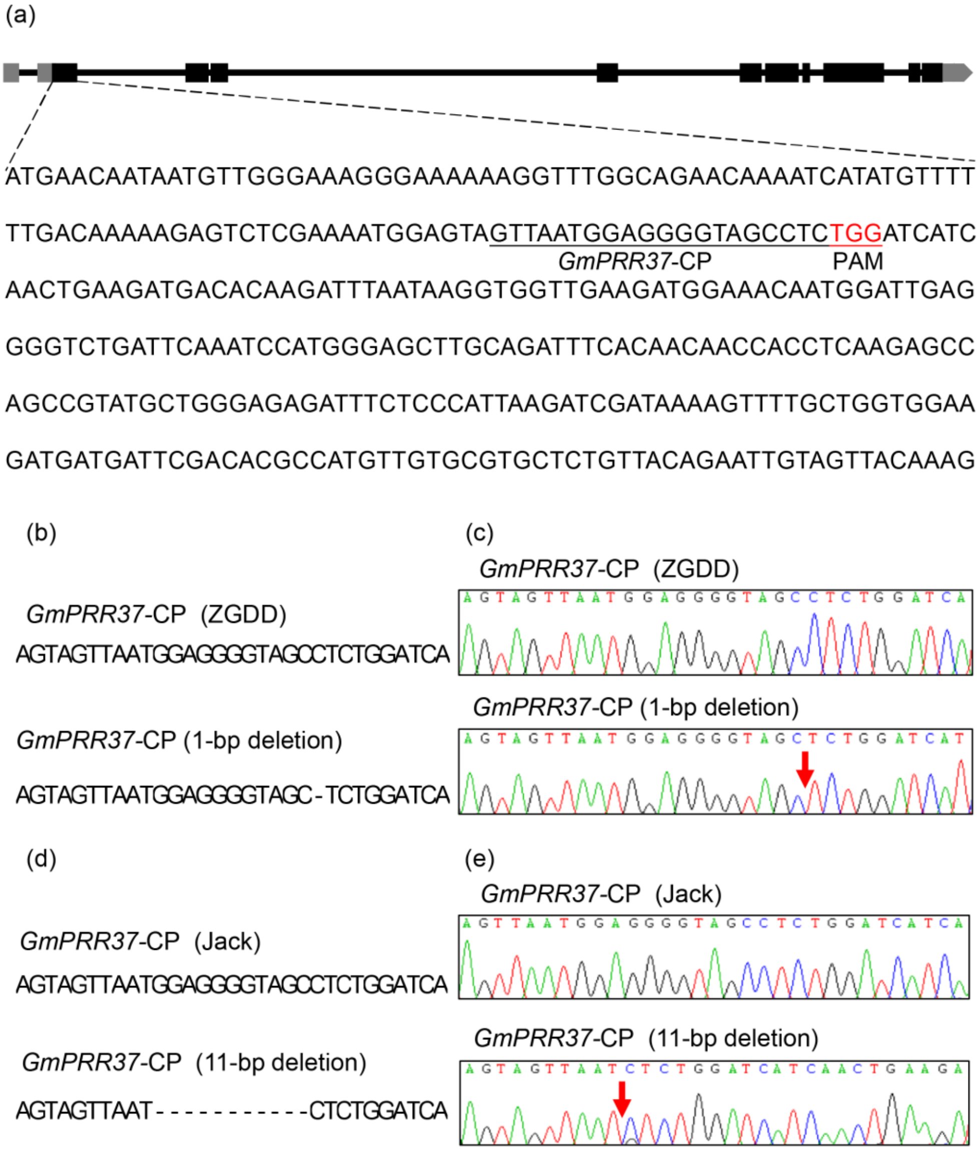

Figure S5 Homozygous targeted mutagenesis of *GmPRR37* (ZGDD) and *Gmprr37* (Jack) induced by CRISPR/Cas9. (a) Gene structures of *GmPRR37* with target sites of CRISPR/Cas9. Grey stripe, 5' UTR. Black line, intron. Black stripe, exon. The underlined nucleotides indicate the target site (named *GmPRR37*-CP). PAM, protospacer adjacent motif. (b) Sequences of WT (ZGDD) and representative mutation type induced at *GmPRR37*-CP is presented. Dashes, deletions. (c) Sequence peaks of WT (ZGDD) and mutation at target site *GmPRR37*-CP. The red arrowhead indicates the location of mutation. (d) Sequences of WT (Jack) and representative mutation type induced at *GmPRR37*-CP is presented. Dashes, deletions. (e) Sequence peaks of WT (Jack) and mutation at target site *GmPRR37*-CP. The red arrowhead indicates the location of mutation.

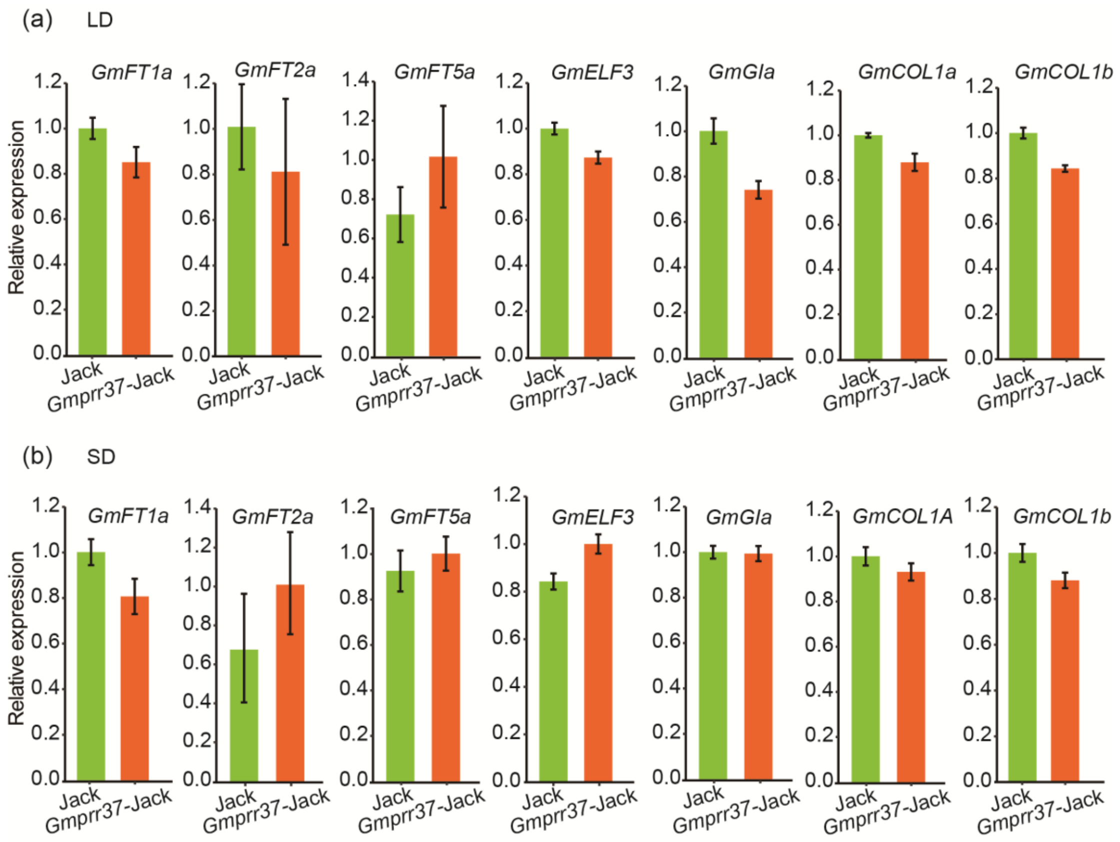

Figure S6 Expression levels of flowering-related genes in leaves of the WT plants (cv Jack) and CRISPR/Cas9-induced *Gmprr37*-Jack mutants under LD and SD conditions. (a) Expression analysis in the WT plants and CRISPR/Cas9-induced *Gmprr37*-Jack mutants under LD (16 h : 8 h, light : dark) conditions. (b) Expression analysis in the WT plants and CRISPR/Cas9-induced *Gmprr37*-Jack mutants under SD (12 h : 12 h, light : dark) conditions.

Table S1 Flowering times of RILs and parents grown under eight different environments and best linear unbiased predictor (BLUP).

Env.	HH27	ZGDD	RIL families						
			Mean	Standard Deviation	Min	Max	Skew	Kurt	*P* value
SY16	17.8	28.6	25.2	3.1	18.4	37.0	0.6	0.5	0.0002
XX16	21.8	68.6	44.1	10.7	18.0	73.8	0.4	0.3	0.0003
JN16	23.4	70.4	47.5	10.7	22.0	73.5	0.1	-0.1	0.0076
BJ16	21.1	90.1	54.6	14.9	21.0	95.8	0.4	0.2	0.0017
SY17	24.1	27.7	25.8	1.6	23.2	32.1	1.1	1.1	0.0001
XT17	22.7	68.7	39.2	10.7	20.6	75.1	1.3	1.7	0.0001
XX17	19.1	79.3	51.9	13.3	18.4	88.9	0.0	0.1	0.0267
BJ17	21.2	97.8	65.2	16.1	21.6	100.5	-0.4	0.1	0.0002
BLUP	.	.	44.2	9.1	22.0	66.8	0.2	0.2	0.0013

HH27: parent inbred line with early flowering; ZGDD: parent inbred line with late flowering time

R1 days from emergency to beginning bloom

Env., represents environment; SY16, XX16, JN16 and BJ16 represent Sanya, Xinxiang, Jining and Beijing in 2016, respectively; SY17, XT17, XX17 and BJ17 represent Sanya, Xiangtan, Xinxiang and Beijing in 2017, respectively

P value, results from the Shapiro-Wilk test for normalized detection

BLUP best linear unbiased predictor, BLUP values of flowering time for each line were obtained across eight environments.

Table S2 Statistics of 2b-RAD reads and mapping rates of the RILs and parents (sequencing of DNA digested with *Bsa*XI).

Sample	Clean data	Enzyme	Percentage	Tag number	Depth	Mapping rate
Heihe27	14,370,646	13,237,201	92.10%	134,242	44.12	44.74%
Zigongdongdou	14,370,646	13,230,324	92.10%	130,067	42.7	41.98%
WL1	5,096,193	4,699,950	92.20%	101,028	20.53	44.13%
WL2	5,096,193	4,689,841	92.00%	115,894	17.7	43.74%
WL3	5,096,193	4,695,204	92.10%	101,742	19.74	42.78%
WL4	5,096,193	4,692,713	92.10%	99,427	20.61	43.67%
WL5	5,096,193	4,454,827	87.40%	112,734	17.85	45.17%
WL6	4,889,926	4,501,438	92.10%	101,096	19.38	43.52%
WL7	4,889,926	4,535,898	92.80%	114,190	17.13	43.12%
WL8	4,889,926	4,535,652	92.80%	98,663	19.66	42.77%
WL9	4,889,926	4,509,217	92.20%	99,784	19.92	44.08%
WL10	4,889,926	4,248,748	86.90%	110,830	16.01	41.76%
WL11	5,376,512	4,892,283	91.00%	106,701	21.33	46.52%
WL12	5,376,512	4,910,081	91.30%	116,734	17.07	40.58%
WL13	5,376,512	4,914,885	91.40%	103,032	20.42	42.81%
WL14	5,376,512	4,890,542	91.00%	103,795	20.96	44.48%
WL15	5,376,512	4,500,658	83.70%	113,393	16.69	42.05%
WL16	5,289,683	4,825,430	91.20%	106,907	19.59	43.40%
WL17	5,289,683	4,829,239	91.30%	116,829	17.36	42.00%
WL18	5,289,683	4,817,227	91.10%	101,426	20.16	42.45%
WL19	5,289,683	4,801,257	90.80%	106,533	20.15	44.71%
WL20	5,289,683	4,571,120	86.40%	111,188	16.67	40.55%
WL21	4,839,940	4,434,641	91.60%	106,908	18.01	43.42%
WL22	4,839,940	4,476,749	92.50%	116,815	16.34	42.64%
WL23	4,839,940	4,509,160	93.20%	102,452	18.96	43.08%
WL24	4,839,940	4,493,948	92.90%	104,618	19.47	45.33%
WL25	4,839,940	4,216,072	87.10%	112,737	16.08	43.00%
WL26	4,727,336	4,339,933	91.80%	102,210	17.74	41.78%
WL27	4,727,336	4,351,823	92.10%	114,567	15.85	41.73%
WL28	4,727,336	4,370,928	92.50%	101,300	19.08	44.22%
WL29	4,727,336	4,332,378	91.60%	101,773	18.65	43.81%
WL30	4,727,336	4,100,555	86.70%	111,951	15.82	43.19%
WL31	4,447,140	4,020,061	90.40%	103,175	17.36	44.55%
WL32	4,447,140	4,070,194	91.50%	110,565	15.34	41.67%
WL33	4,447,140	4,022,209	90.40%	96,680	16.64	40.00%
WL34	4,447,140	4,041,593	90.90%	99,446	18	44.29%
WL35	4,447,140	3,832,427	86.20%	107,095	14.28	39.90%
WL36	5,158,998	4,509,110	87.40%	101,925	18.55	41.93%
WL37	5,158,998	4,535,348	87.90%	114,599	16.96	42.85%

WL38	5,158,998	4,501,943	87.30%	101,967	19.3	43.71%
WL39	5,158,998	4,481,881	86.90%	104,051	19.68	45.69%
WL40	5,158,998	4,230,151	82.00%	109,912	15.96	41.47%
WL41	4,731,990	3,503,352	74.00%	115,020	14.13	46.39%
WL42	4,731,990	4,458,233	94.20%	120,363	15.65	42.25%
WL43	4,731,990	4,476,730	94.60%	116,650	16.8	43.78%
WL44	4,731,990	4,388,277	92.70%	120,508	16.3	44.76%
WL45	4,731,990	3,356,445	70.90%	124,071	12.19	45.06%
WL46	4,369,405	3,227,144	73.90%	109,689	13.11	44.56%
WL47	4,369,405	4,047,166	92.60%	119,751	14.61	43.23%
WL48	4,369,405	4,079,830	93.40%	115,838	14.79	41.99%
WL49	4,369,405	4,015,312	91.90%	121,635	15.94	48.29%
WL50	4,369,405	3,009,254	68.90%	119,759	10.75	42.78%
WL51	4,891,789	3,638,251	74.40%	112,678	14.52	44.97%
WL52	4,891,789	4,510,023	92.20%	121,769	15.33	41.39%
WL53	4,891,789	4,544,657	92.90%	120,091	16.95	44.79%
WL54	4,891,789	4,531,591	92.60%	121,302	17.53	46.92%
WL55	4,891,789	3,329,828	68.10%	122,727	12.19	44.93%
WL56	5,525,593	4,097,286	74.20%	115,604	15.47	43.65%
WL57	5,525,593	5,158,849	93.40%	124,328	17.71	42.68%
WL58	5,525,593	5,158,420	93.40%	123,333	19.18	45.86%
WL59	5,525,593	5,115,054	92.60%	124,682	19.32	47.09%
WL60	5,525,593	3,708,160	67.10%	124,014	12.98	43.41%
WL61	7,453,329	5,601,126	75.10%	122,449	20.87	45.62%
WL62	7,453,329	6,963,571	93.40%	130,462	25.56	47.89%
WL63	7,453,329	6,987,931	93.80%	126,373	23.38	42.28%
WL64	7,453,329	6,921,439	92.90%	128,014	24.22	44.80%
WL65	7,453,329	4,870,147	65.30%	132,470	16.63	45.23%
WL66	4,749,263	3,524,289	74.20%	111,909	14.55	46.20%
WL67	4,749,263	4,350,688	91.60%	123,155	16.58	46.93%
WL68	4,749,263	4,421,151	93.10%	118,401	16.38	43.87%
WL69	4,749,263	4,349,653	91.60%	121,311	16.54	46.13%
WL70	4,749,263	3,240,307	68.20%	123,250	11.42	43.44%
WL71	6,593,863	4,904,594	74.40%	119,544	18.59	45.31%
WL72	6,593,863	6,111,779	92.70%	128,595	22.19	46.69%
WL73	6,593,863	6,037,763	91.60%	126,466	20.72	43.40%
WL74	6,593,863	5,938,255	90.10%	129,060	20.55	44.66%
WL75	6,593,863	4,324,916	65.60%	131,064	14.73	44.64%
WL76	5,265,925	3,869,883	73.50%	117,095	15.18	45.93%
WL77	5,265,925	4,740,080	90.00%	126,218	17.37	46.25%
WL78	5,265,925	4,759,319	90.40%	122,794	17.27	44.56%
WL79	5,265,925	4,822,899	91.60%	125,281	18.5	48.06%
WL80	5,265,925	3,405,342	64.70%	125,579	12.04	44.40%
WL81	5,098,010	3,783,948	74.20%	115,134	14.62	44.48%

WL82	5,098,010	4,700,122	92.20%	125,185	17.97	47.86%
WL83	5,098,010	4,691,732	92.00%	120,996	16.34	42.14%
WL84	5,098,010	4,664,172	91.50%	124,331	17.26	46.01%
WL85	5,098,010	3,519,987	69.00%	123,587	12.32	43.26%
WL86	4,199,999	3,028,118	72.10%	109,782	11.5	41.69%
WL87	4,199,999	3,766,377	89.70%	120,614	14.18	45.41%
WL88	4,199,999	3,748,097	89.20%	117,997	13.65	42.97%
WL89	4,199,999	3,859,048	91.90%	118,519	14.89	45.73%
WL90	4,199,999	3,041,470	72.40%	124,112	11.6	47.34%
WL91	4,886,127	3,553,815	72.70%	114,833	13.26	42.85%
WL92	4,886,127	4,412,698	90.30%	123,792	16.39	45.98%
WL93	4,886,127	4,493,371	92.00%	119,951	15.67	41.83%
WL94	4,886,127	4,438,120	90.80%	122,500	17.35	47.89%
WL95	4,886,127	3,430,799	70.20%	124,213	11.64	42.14%
WL96	3,621,934	2,669,497	73.70%	106,302	10.91	43.44%
WL97	3,621,934	3,319,955	91.70%	116,118	12.6	44.07%
WL98	3,621,934	3,261,895	90.10%	115,435	12.52	44.31%
WL99	3,621,934	3,336,575	92.10%	113,685	12.77	43.51%
WL100	3,621,934	2,612,869	72.10%	119,455	9.71	44.39%
WL101	5,436,455	3,928,859	72.30%	113,035	15.79	45.43%
WL102	5,436,455	4,793,943	88.20%	125,737	17.05	44.72%
WL103	5,436,455	4,926,829	90.60%	119,538	18.26	44.30%
WL104	5,436,455	4,949,054	91.00%	121,187	18.73	45.86%
WL105	5,436,455	3,423,571	63.00%	121,453	11.65	41.33%
WL106	5,914,297	4,139,680	70.00%	119,548	15.94	46.03%
WL107	5,914,297	5,427,558	91.80%	125,480	18.98	43.88%
WL108	5,914,297	5,424,569	91.70%	121,941	19.27	43.32%
WL109	5,914,297	5,302,887	89.70%	124,821	18.68	43.97%
WL110	5,914,297	4,101,832	69.40%	127,435	14.29	44.40%
WL111	4,030,105	2,657,885	66.00%	103,916	11.18	43.71%
WL112	4,030,105	3,585,648	89.00%	118,248	13.62	44.92%
WL113	4,030,105	3,598,854	89.30%	115,557	14.01	44.99%
WL114	4,030,105	3,524,568	87.50%	116,114	13.87	45.69%
WL115	4,030,105	1,848,858	45.90%	103,591	7.69	43.09%
WL116	5,511,038	4,043,215	73.40%	112,611	15.59	43.42%
WL117	5,511,038	5,034,020	91.30%	123,957	17.97	44.25%
WL118	5,511,038	5,040,924	91.50%	119,281	18.43	43.61%
WL119	5,511,038	4,992,528	90.60%	122,882	18.89	46.49%
WL120	5,511,038	3,885,359	70.50%	127,663	13.71	45.05%
WL121	4,310,758	2,919,656	67.70%	107,146	12.47	45.76%
WL122	4,310,758	3,927,672	91.10%	118,616	15.16	45.78%
WL123	4,310,758	3,977,733	92.30%	109,732	15.58	42.98%
WL124	4,310,758	3,927,678	91.10%	114,195	15.73	45.73%
WL125	4,310,758	1,920,660	44.60%	105,100	8.01	43.83%

WL126	3,871,117	2,618,710	67.60%	106,450	11.47	46.63%
WL127	3,871,117	3,534,407	91.30%	115,834	13.42	43.98%
WL128	3,871,117	3,582,932	92.60%	108,927	14.06	42.74%
WL129	3,871,117	3,536,472	91.40%	113,081	15.2	48.60%
WL130	3,871,117	1,831,463	47.30%	103,048	7.98	44.90%
WL131	4,652,740	3,093,874	66.50%	112,666	13.1	47.70%
WL132	4,652,740	4,162,157	89.50%	122,066	15.33	44.96%
WL133	4,652,740	4,265,330	91.70%	114,407	17.09	45.84%
WL134	4,652,740	4,207,564	90.40%	115,809	16.06	44.20%
WL135	4,652,740	2,102,511	45.20%	106,132	8.39	42.35%
WL136	4,053,001	2,686,399	66.30%	107,961	11.09	44.57%
WL137	4,053,001	3,639,665	89.80%	117,574	13.84	44.71%
WL138	4,053,001	3,685,350	90.90%	111,698	14.17	42.95%
WL139	4,053,001	3,632,194	89.60%	115,521	14.47	46.02%
WL140	4,053,001	1,834,181	45.30%	99,787	7.56	41.13%
WL141	3,995,066	2,612,159	65.40%	109,762	11.01	46.26%
WL142	3,995,066	3,548,590	88.80%	116,639	13.28	43.65%
WL143	3,995,066	3,613,430	90.40%	111,257	13.93	42.89%
WL144	3,995,066	3,564,495	89.20%	113,171	13.68	43.43%
WL145	3,995,066	1,791,264	44.80%	101,292	7.38	41.73%
WL146	5,770,944	3,822,764	66.20%	114,768	14.7	44.13%
WL147	5,770,944	5,129,974	88.90%	125,718	19.08	46.76%
WL148	5,770,944	5,250,323	91.00%	118,829	19.43	43.98%
WL149	5,770,944	5,198,955	90.10%	120,499	20.02	46.40%
WL150	5,770,944	2,707,994	46.90%	118,616	10.49	45.95%
WL151	3,757,069	2,485,725	66.20%	106,595	10.98	47.09%
WL152	3,757,069	3,357,429	89.40%	114,420	12.7	43.28%
WL153	3,757,069	3,412,261	90.80%	107,693	13.46	42.48%
WL154	3,757,069	3,397,026	90.40%	109,329	13.99	45.03%
WL155	3,757,069	1,657,473	44.10%	100,716	7.35	44.66%
WL156	2,653,733	1,726,266	65.10%	91,490	8.36	44.31%
WL157	2,653,733	2,376,812	89.60%	103,922	10	43.72%
WL158	2,653,733	2,361,014	89.00%	99,352	10.25	43.13%
WL159	2,653,733	2,391,650	90.10%	97,198	10.83	44.01%
WL160	2,653,733	1,181,649	44.50%	81,150	6.16	42.30%
WL161	3,737,022	2,477,061	66.30%	104,880	10.75	45.52%
WL162	3,737,022	3,373,423	90.30%	116,515	13.89	47.97%
WL163	3,737,022	3,412,130	91.30%	107,965	13.37	42.30%
WL164	3,737,022	3,371,965	90.20%	113,195	14.31	48.04%
WL165	3,737,022	1,665,356	44.60%	99,365	7.12	42.48%
WL166	4,859,838	3,263,266	67.10%	109,356	12.56	42.09%
WL167	4,859,838	4,380,331	90.10%	122,005	16.68	46.46%
WL168	4,859,838	4,439,367	91.30%	115,633	17.27	44.98%
WL169	4,859,838	4,432,852	91.20%	115,658	17.15	44.75%

WL170	4,859,838	2,352,376	48.40%	113,219	9.26	44.57%
WL171	4,207,312	2,809,694	66.80%	110,009	11.3	44.24%
WL172	4,207,312	3,827,690	91.00%	118,269	14.61	45.14%
WL173	4,207,312	3,888,335	92.40%	111,889	15.77	45.38%
WL174	4,207,312	3,841,222	91.30%	115,002	15.73	47.09%
WL175	4,207,312	2,019,189	48.00%	104,594	8.19	42.42%
WL176	4,897,031	3,235,831	66.10%	115,247	12.89	45.91%
WL177	4,897,031	4,385,673	89.60%	120,783	15.55	42.83%
WL178	4,897,031	4,453,240	90.90%	114,192	16.79	43.05%
WL179	4,897,031	4,365,497	89.10%	119,792	17.13	47.01%
WL180	4,897,031	2,315,872	47.30%	112,801	9.52	46.37%
WL181	4,239,058	2,858,938	67.40%	102,785	12.02	43.21%
WL182	4,239,058	3,857,540	91.00%	119,395	14.4	44.57%
WL183	4,239,058	3,880,048	91.50%	111,807	15.71	45.27%
WL184	4,239,058	3,879,588	91.50%	114,145	15.82	46.55%
WL185	4,239,058	2,127,068	50.20%	107,585	8.5	42.99%
WL186	4,889,202	3,254,987	66.60%	110,483	12.91	43.82%
WL187	4,889,202	4,416,791	90.30%	121,327	17.11	47.00%
WL188	4,889,202	4,492,476	91.90%	113,788	17.71	44.86%
WL189	4,889,202	4,453,313	91.10%	117,744	17.91	47.35%
WL190	4,889,202	2,347,674	48.00%	110,545	9.3	43.79%
WL191	4,013,844	2,720,843	67.80%	101,201	11.42	42.48%
WL192	4,013,844	3,629,472	90.40%	118,902	14.6	47.83%
WL193	4,013,844	3,698,155	92.10%	107,910	14.57	42.51%
WL194	4,013,844	3,642,496	90.70%	113,801	15.14	47.30%
WL195	4,013,844	1,794,165	44.70%	102,438	7.65	43.68%
WL196	4,469,902	3,004,433	67.20%	105,631	12.37	43.49%
WL197	4,469,902	4,036,616	90.30%	120,624	15.17	45.33%
WL198	4,469,902	4,105,285	91.80%	108,953	15.86	42.09%
WL199	4,469,902	4,070,162	91.10%	113,044	16.19	44.97%
WL200	4,469,902	2,174,987	48.70%	111,432	9.02	46.21%
WL201	6,454,845	4,414,731	68.40%	107,993	18.78	45.94%
WL202	6,454,845	5,895,393	91.30%	128,673	21.27	46.42%
WL203	6,454,845	5,986,662	92.70%	111,862	23.25	43.44%
WL204	6,454,845	5,923,188	91.80%	108,127	24.16	44.10%
WL205	6,454,845	3,021,482	46.80%	110,724	12.24	44.85%
WL206	4,300,750	2,933,007	68.20%	93,727	14.3	45.70%
WL207	4,300,750	3,904,784	90.80%	121,395	14.38	44.71%
WL208	4,300,750	3,955,295	92.00%	104,912	16.73	44.38%
WL209	4,300,750	3,927,418	91.30%	98,242	17.8	44.53%
WL210	4,300,750	1,972,374	45.90%	93,480	8.79	41.66%
WL211	4,520,193	3,062,070	67.70%	96,883	14.29	45.21%
WL212	4,520,193	4,109,523	90.90%	121,643	14.82	43.87%
WL213	4,520,193	4,143,166	91.70%	105,351	17.5	44.50%

WL214	4,520,193	4,120,197	91.20%	98,273	18.65	44.48%
WL215	4,520,193	2,122,438	47.00%	94,327	9.41	41.82%
WL216	4,539,310	3,106,162	68.40%	98,454	14.14	44.82%
WL217	4,539,310	4,165,422	91.80%	119,096	14.02	40.09%
WL218	4,539,310	4,193,891	92.40%	106,726	17.28	43.97%
WL219	4,539,310	4,189,140	92.30%	97,155	19.33	44.83%
WL220	4,539,310	2,261,111	49.80%	100,478	9.4	41.77%
WL221	5,396,314	3,589,961	66.50%	103,979	15.87	45.97%
WL222	5,396,314	4,822,841	89.40%	126,624	17.07	44.82%
WL223	5,396,314	4,927,043	91.30%	107,781	19.51	42.68%
WL224	5,396,314	4,853,233	89.90%	107,509	20.68	45.81%
WL225	5,396,314	2,548,204	47.20%	103,816	9.99	40.70%
WL226	5,815,533	3,893,957	67.00%	109,581	16.4	46.15%
WL227	5,815,533	5,256,647	90.40%	125,900	17.9	42.87%
WL228	5,815,533	5,347,617	92.00%	107,976	20.75	41.90%
WL229	5,815,533	5,255,465	90.40%	105,077	22.35	44.69%
WL230	5,815,533	2,725,514	46.90%	106,378	10.79	42.11%
WL231	4,501,260	3,035,064	67.40%	96,929	14.03	44.81%
WL232	4,501,260	4,078,806	90.60%	121,852	14.69	43.89%
WL233	4,501,260	4,132,034	91.80%	104,575	17.12	43.33%
WL234	4,501,260	4,108,782	91.30%	96,721	19.15	45.08%
WL235	4,501,260	2,041,100	45.30%	93,963	8.93	41.11%
WL236	3,476,842	2,332,416	67.10%	91,218	11.19	43.76%
WL237	3,476,842	3,143,957	90.40%	114,523	11.72	42.69%
WL238	3,476,842	3,205,756	92.20%	92,804	13.23	38.30%
WL239	3,476,842	3,156,039	90.80%	90,689	15.38	44.19%
WL240	3,476,842	1,670,254	48.00%	86,465	7.81	40.43%
WL241	4,420,678	2,953,867	66.80%	92,961	13.68	43.05%
WL242	4,420,678	3,938,503	89.10%	121,749	14.96	46.25%
WL243	4,420,678	4,011,152	90.70%	104,777	17.24	45.03%
WL244	4,420,678	3,979,667	90.00%	95,723	18.24	43.87%
WL245	4,420,678	1,954,669	44.20%	94,408	8.52	41.15%
WL246	4,495,813	3,013,238	67.00%	95,327	13.76	43.53%
WL247	4,495,813	4,048,375	90.00%	122,136	14.21	42.87%
WL248	4,495,813	4,098,827	91.20%	107,820	17.21	45.27%
WL249	4,495,813	4,059,068	90.30%	101,363	19.02	47.50%
WL250	4,495,813	2,130,384	47.40%	98,110	9.39	43.24%
WL251	3,995,625	2,690,830	67.30%	92,927	12.69	43.82%
WL252	3,995,625	3,592,672	89.90%	117,333	13.71	44.78%
WL253	3,995,625	3,658,637	91.60%	101,456	15.85	43.95%
WL254	3,995,625	3,586,223	89.80%	89,601	18.5	46.22%
WL255	3,995,625	1,842,325	46.10%	92,210	8.54	42.74%
WL256	4,264,021	2,853,504	66.90%	97,351	13.18	44.97%
WL257	4,264,021	3,790,732	88.90%	121,943	13.83	44.49%

WL258	4,264,021	3,859,105	90.50%	105,317	16.21	44.24%
WL259	4,264,021	3,845,485	90.20%	94,395	17.32	42.52%
WL260	4,264,021	2,084,098	48.90%	100,349	9.08	43.72%
WL261	4,311,071	2,879,526	66.80%	96,906	13.47	45.33%
WL262	4,311,071	3,895,125	90.40%	119,188	13.82	42.29%
WL263	4,311,071	3,918,412	90.90%	102,782	15.65	41.05%
WL264	4,311,071	3,903,412	90.50%	94,371	17.8	43.03%
WL265	4,311,071	2,069,568	48.00%	97,370	9.13	42.96%
WL266	4,203,990	2,829,711	67.30%	97,660	13.49	46.56%
WL267	4,203,990	3,808,489	90.60%	119,737	13.36	42.00%
WL268	4,203,990	3,853,318	91.70%	105,262	16.47	44.99%
WL269	4,203,990	3,816,043	90.80%	94,677	17.26	42.82%
WL270	4,203,990	2,168,033	51.60%	96,133	8.97	39.77%
WL271	3,962,436	2,651,012	66.90%	89,030	13.14	44.13%
WL272	3,962,436	3,558,263	89.80%	117,839	13.3	44.05%
WL273	3,962,436	3,608,295	91.10%	98,552	15.5	42.33%
WL274	3,962,436	3,562,561	89.90%	94,198	17.1	45.21%
WL275	3,962,436	1,945,859	49.10%	92,729	8.6	40.98%
WL276	4,769,700	3,192,969	66.90%	98,920	14.34	44.43%
WL277	4,769,700	4,271,502	89.60%	122,879	15.7	45.16%
WL278	4,769,700	4,293,469	90.00%	108,825	17.57	44.53%
WL279	4,769,700	4,309,397	90.30%	98,139	18.92	43.09%
WL280	4,769,700	2,443,280	51.20%	103,281	10.01	42.31%
WL281	4,312,916	3,163,253	73.30%	107,539	13.05	44.37%
WL282	4,312,916	3,930,853	91.10%	118,098	14.01	42.09%
WL283	4,312,916	3,980,599	92.30%	108,820	16.3	44.56%
WL284	4,312,916	3,941,697	91.40%	113,125	14.69	42.16%
WL285	4,312,916	2,042,012	47.30%	103,000	8.52	42.98%
WL286	5,295,828	3,894,006	73.50%	116,192	15.39	45.92%
WL287	5,295,828	4,871,907	92.00%	124,614	18.38	47.01%
WL288	5,295,828	4,904,846	92.60%	112,416	17.86	40.93%
WL289	5,295,828	4,909,788	92.70%	117,208	18.83	44.95%
WL290	5,295,828	3,802,201	71.80%	123,189	13.27	42.99%
WL291	4,916,586	3,617,364	73.60%	110,701	13.69	41.90%
WL292	4,916,586	4,574,174	93.00%	122,387	16.74	44.79%
WL293	4,916,586	4,556,403	92.70%	112,548	17.43	43.05%
WL294	4,916,586	4,501,442	91.60%	119,716	17.2	45.74%
WL295	4,916,586	3,531,594	71.80%	121,931	12.69	43.81%
WL296	3,879,408	2,841,322	73.20%	101,776	12.61	45.17%
WL297	3,879,408	3,542,662	91.30%	113,375	12.41	39.72%
WL298	3,879,408	3,578,410	92.20%	104,604	15.05	43.99%
WL299	3,879,408	3,557,991	91.70%	109,505	14.88	45.80%
WL300	3,879,408	1,854,418	47.80%	95,165	7.84	40.23%
WL301	4,376,801	3,197,988	73.10%	107,955	12.47	42.10%

WL302	4,376,801	4,024,582	92.00%	120,642	15.27	45.77%
WL303	4,376,801	4,080,507	93.20%	106,195	16.52	42.99%
WL304	4,376,801	4,002,957	91.50%	115,448	15.98	46.09%
WL305	4,376,801	2,088,472	47.70%	101,103	8.5	41.15%
WL306	14,370,646	10,515,324	73.20%	131,351	37.01	46.23%
WL307	14,370,646	13,257,299	92.30%	134,176	44.82	45.36%
WL308	14,370,646	6,962,191	48.40%	131,259	23.74	44.76%

Table S3 Statistics of 2b-RAD reads and mapping rates of the RILs and parents (sequencing of DNA digested with *Fal*I).

Sample	Clean data	Enzyme	Percentage	Tag number	Depth	Mapping rate
Heihe27	39,503,519	35,862,679	90.80%	189,174	92.59	48.84%
Zigongdongdou	39,503,519	35,952,363	91.00%	188,114	89.57	46.87%
WL1	12,451,205	11,073,568	88.90%	158,493	32	45.80%
WL2	12,451,205	11,273,988	90.50%	161,800	32.78	47.04%
WL3	12,451,205	11,361,582	91.20%	161,841	33.13	47.19%
WL4	12,451,205	11,330,816	91.00%	164,844	32.76	47.66%
WL5	12,451,205	11,047,087	88.70%	174,147	32.55	51.31%
WL6	15,276,992	13,478,252	88.20%	162,384	37.41	45.07%
WL7	15,276,992	13,736,201	89.90%	166,799	39.54	48.01%
WL8	15,276,992	13,841,047	90.60%	168,540	38.58	46.98%
WL9	15,276,992	13,804,340	90.40%	172,483	39.39	49.22%
WL10	15,276,992	13,443,466	88.00%	177,504	35.6	47.01%
WL11	9,998,653	9,295,840	93.00%	161,398	28.09	48.77%
WL12	9,998,653	9,260,042	92.60%	157,448	26.11	44.39%
WL13	9,998,653	9,293,381	92.90%	161,899	26.61	46.36%
WL14	9,998,653	9,243,065	92.40%	179,471	25.42	49.36%
WL15	9,998,653	8,773,364	87.70%	169,420	23.85	46.06%
WL16	8,933,255	8,517,992	95.40%	176,462	21.42	44.37%
WL17	8,933,255	8,508,497	95.20%	177,934	21.52	45.00%
WL18	8,933,255	8,527,263	95.50%	177,983	22.18	46.29%
WL19	8,933,255	8,521,956	95.40%	176,003	23.97	49.50%
WL20	8,933,255	8,478,198	94.90%	181,269	21.92	46.87%
WL21	7,971,068	7,561,404	94.90%	180,183	19.69	46.92%
WL22	7,971,068	7,553,269	94.80%	176,895	19.85	46.49%
WL23	7,971,068	7,564,370	94.90%	176,410	19.91	46.43%
WL24	7,971,068	7,568,020	94.90%	173,963	21.88	50.29%
WL25	7,971,068	7,527,221	94.40%	182,236	20.51	49.66%
WL26	6,474,911	6,147,195	94.90%	170,346	15.64	43.34%
WL27	6,474,911	6,141,191	94.80%	171,690	16.41	45.88%
WL28	6,474,911	6,154,507	95.10%	180,056	17.63	51.58%
WL29	6,474,911	6,161,879	95.20%	165,863	19.2	51.68%
WL30	6,474,911	6,118,858	94.50%	176,899	17.49	50.56%
WL31	7,425,356	7,081,124	95.40%	175,868	19.74	49.03%
WL32	7,425,356	7,060,579	95.10%	173,584	18.6	45.73%
WL33	7,425,356	7,091,198	95.50%	175,579	18.23	45.14%
WL34	7,425,356	7,103,267	95.70%	171,464	20.73	50.04%
WL35	7,425,356	7,057,513	95.00%	177,248	18.16	45.61%
WL36	6,968,991	6,640,255	95.30%	171,513	16.54	42.72%
WL37	6,968,991	6,639,656	95.30%	172,080	17.98	46.60%

WL38	6,968,991	6,652,098	95.50%	175,905	18.27	48.31%
WL39	6,968,991	6,648,940	95.40%	171,461	20.39	52.58%
WL40	6,968,991	6,613,216	94.90%	177,292	17.41	46.67%
WL41	6,554,564	6,222,679	94.90%	169,753	17.76	48.45%
WL42	6,554,564	6,227,309	95.00%	166,342	16.63	44.42%
WL43	6,554,564	6,241,668	95.20%	170,854	18.04	49.38%
WL44	6,554,564	6,237,271	95.20%	165,741	19.04	50.59%
WL45	6,554,564	6,196,880	94.50%	175,560	17.47	49.49%
WL46	5,744,404	5,394,896	93.90%	173,236	14.27	45.82%
WL47	5,744,404	5,402,207	94.00%	170,506	14.18	44.76%
WL48	5,744,404	5,413,818	94.20%	172,526	14.28	45.51%
WL49	5,744,404	5,411,771	94.20%	172,238	16.48	52.45%
WL50	5,744,404	5,373,353	93.50%	176,024	14.08	46.12%
WL51	6,305,510	5,946,821	94.30%	175,678	15.65	46.23%
WL52	6,305,510	5,949,018	94.30%	168,903	15.02	42.64%
WL53	6,305,510	5,965,650	94.60%	174,695	16.53	48.41%
WL54	6,305,510	5,963,820	94.60%	173,298	17.59	51.11%
WL55	6,305,510	5,921,513	93.90%	178,039	16.05	48.26%
WL56	6,392,012	6,041,642	94.50%	175,024	15.16	43.92%
WL57	6,392,012	6,041,056	94.50%	171,738	15.46	43.95%
WL58	6,392,012	6,056,492	94.80%	176,702	16.86	49.19%
WL59	6,392,012	6,055,399	94.70%	174,377	17.7	50.97%
WL60	6,392,012	6,017,229	94.10%	177,966	15.61	46.17%
WL61	10,071,332	9,512,680	94.50%	185,077	23.79	46.29%
WL62	10,071,332	9,505,754	94.40%	185,105	24.97	48.62%
WL63	10,071,332	9,536,402	94.70%	184,594	22.97	44.46%
WL64	10,071,332	9,531,700	94.60%	181,536	24.95	47.52%
WL65	10,071,332	9,444,576	93.80%	188,347	25.12	50.10%
WL66	9,460,716	8,841,062	93.50%	184,844	23.16	48.42%
WL67	9,460,716	8,811,935	93.10%	183,455	23.48	48.88%
WL68	9,460,716	8,833,787	93.40%	180,937	22.85	46.80%
WL69	9,460,716	8,809,989	93.10%	180,305	24.49	50.12%
WL70	9,460,716	8,399,556	88.80%	183,830	21.29	46.59%
WL71	10,817,195	10,021,605	92.60%	185,225	25.32	46.80%
WL72	10,817,195	9,978,884	92.30%	184,666	25.75	47.65%
WL73	10,817,195	10,020,112	92.60%	185,408	25.34	46.89%
WL74	10,817,195	10,009,310	92.50%	182,284	27.02	49.21%
WL75	10,817,195	9,468,232	87.50%	187,137	24.92	49.25%
WL76	10,817,158	10,059,307	93.00%	189,088	26.42	49.66%
WL77	10,817,158	10,031,651	92.70%	189,415	25.85	48.81%
WL78	10,817,158	10,058,182	93.00%	187,427	27.24	50.76%
WL79	10,817,158	10,045,762	92.90%	184,649	28.59	52.55%
WL80	10,817,158	9,539,286	88.20%	188,660	24.64	48.73%
WL81	9,518,979	8,769,958	92.10%	185,134	21.49	45.37%

WL82	9,518,979	8,735,408	91.80%	185,503	23.19	49.25%
WL83	9,518,979	8,764,392	92.10%	181,342	21.51	44.51%
WL84	9,518,979	8,755,313	92.00%	180,382	23.91	49.26%
WL85	9,518,979	8,201,475	86.20%	183,045	20.75	46.31%
WL86	14,099,448	13,042,138	92.50%	186,182	30.56	43.63%
WL87	14,099,448	12,997,611	92.20%	188,621	33.35	48.40%
WL88	14,099,448	13,021,226	92.40%	186,496	32.75	46.91%
WL89	14,099,448	13,006,582	92.20%	184,589	34.43	48.86%
WL90	14,099,448	12,288,830	87.20%	189,798	32.89	50.80%
WL91	11,354,354	10,405,585	91.60%	185,850	25.1	44.83%
WL92	11,354,354	10,387,136	91.50%	188,987	26.47	48.16%
WL93	11,354,354	10,415,587	91.70%	188,173	26.04	47.05%
WL94	11,354,354	10,403,201	91.60%	183,540	30.59	53.97%
WL95	11,354,354	9,857,941	86.80%	184,786	24.28	45.51%
WL96	10,399,450	9,838,974	94.60%	185,467	24.19	45.60%
WL97	10,399,450	9,825,464	94.50%	186,490	25.11	47.66%
WL98	10,399,450	9,837,117	94.60%	187,315	26.72	50.88%
WL99	10,399,450	9,841,250	94.60%	182,780	25.75	47.83%
WL100	10,399,450	9,773,338	94.00%	187,549	25.65	49.22%
WL101	12,522,728	11,790,786	94.20%	188,420	30.17	48.21%
WL102	12,522,728	11,766,793	94.00%	187,820	30.76	49.10%
WL103	12,522,728	11,806,756	94.30%	188,030	29.89	47.60%
WL104	12,522,728	11,808,299	94.30%	185,488	31.12	48.88%
WL105	12,522,728	11,715,588	93.60%	185,507	27.66	43.80%
WL106	12,630,501	11,900,584	94.20%	188,760	31.7	50.28%
WL107	12,630,501	11,899,707	94.20%	186,276	29.28	45.83%
WL108	12,630,501	11,940,658	94.50%	187,181	29.76	46.65%
WL109	12,630,501	11,931,952	94.50%	185,330	30.75	47.76%
WL110	12,630,501	11,847,544	93.80%	189,419	29.72	47.52%
WL111	11,684,625	11,054,460	94.60%	188,641	27.96	47.71%
WL112	11,684,625	11,035,805	94.40%	183,438	27.49	45.69%
WL113	11,684,625	11,059,190	94.60%	187,805	29.46	50.03%
WL114	11,684,625	11,061,518	94.70%	180,191	30.65	49.93%
WL115	11,684,625	10,961,663	93.80%	186,403	28.24	48.02%
WL116	10,461,847	9,869,445	94.30%	186,534	26.02	49.18%
WL117	10,461,847	9,842,138	94.10%	182,693	25.15	46.68%
WL118	10,461,847	9,872,928	94.40%	182,715	25.7	47.56%
WL119	10,461,847	9,872,491	94.40%	182,705	27.01	49.99%
WL120	10,461,847	9,790,473	93.60%	186,955	25.35	48.41%
WL121	10,389,359	9,771,987	94.10%	185,505	25.41	48.24%
WL122	10,389,359	9,753,483	93.90%	180,523	24.84	45.98%
WL123	10,389,359	9,778,692	94.10%	181,289	25.27	46.85%
WL124	10,389,359	9,794,188	94.30%	180,169	26.53	48.80%
WL125	10,389,359	9,690,020	93.30%	186,468	25.31	48.70%

WL126	12,968,780	12,281,586	94.70%	189,200	32.05	49.37%
WL127	12,968,780	12,267,583	94.60%	186,181	29.48	44.74%
WL128	12,968,780	12,298,794	94.80%	185,837	31.18	47.11%
WL129	12,968,780	12,300,375	94.80%	186,007	34.66	52.41%
WL130	12,968,780	12,193,056	94.00%	186,862	33.53	51.39%
WL131	11,788,718	11,081,162	94.00%	186,402	30.22	50.83%
WL132	11,788,718	11,066,038	93.90%	186,065	27.4	46.07%
WL133	11,788,718	11,112,508	94.30%	187,178	30.09	50.68%
WL134	11,788,718	11,114,848	94.30%	182,843	28.94	47.61%
WL135	11,788,718	11,011,683	93.40%	184,487	28.2	47.25%
WL136	9,355,962	8,787,836	93.90%	182,817	21.99	45.75%
WL137	9,355,962	8,775,625	93.80%	180,120	22.47	46.12%
WL138	9,355,962	8,816,651	94.20%	183,393	22.53	46.86%
WL139	9,355,962	8,812,662	94.20%	179,986	24	49.02%
WL140	9,355,962	8,748,921	93.50%	181,976	22.01	45.78%
WL141	10,899,535	10,288,488	94.40%	186,799	26.52	48.15%
WL142	10,899,535	10,272,802	94.20%	181,738	25.41	44.95%
WL143	10,899,535	10,312,726	94.60%	185,248	25.98	46.67%
WL144	10,899,535	10,304,942	94.50%	179,806	27.53	48.04%
WL145	10,899,535	10,214,362	93.70%	183,338	26.14	46.92%
WL146	11,683,321	10,836,041	92.70%	183,981	27.52	46.73%
WL147	11,683,321	10,801,079	92.40%	185,633	28.72	49.36%
WL148	11,683,321	10,836,787	92.80%	184,917	28.42	48.50%
WL149	11,683,321	10,828,320	92.70%	180,299	30.25	50.37%
WL150	11,683,321	10,278,429	88.00%	186,182	28.03	50.77%
WL151	10,834,901	9,979,088	92.10%	185,081	27.74	51.45%
WL152	10,834,901	9,943,148	91.80%	181,699	25.32	46.27%
WL153	10,834,901	9,965,537	92.00%	181,557	26.84	48.90%
WL154	10,834,901	9,948,811	91.80%	178,614	27.1	48.65%
WL155	10,834,901	9,416,785	86.90%	185,407	25.65	50.50%
WL156	11,971,587	11,090,705	92.60%	186,865	29.21	49.22%
WL157	11,971,587	11,070,569	92.50%	185,614	27.58	46.24%
WL158	11,971,587	11,088,067	92.60%	186,039	29.77	49.95%
WL159	11,971,587	11,092,883	92.70%	181,794	31.02	50.84%
WL160	11,971,587	10,537,652	88.00%	187,262	28.83	51.23%
WL161	10,695,843	9,960,008	93.10%	185,331	26.08	48.53%
WL162	10,695,843	9,923,996	92.80%	183,684	26.92	49.83%
WL163	10,695,843	9,950,811	93.00%	182,703	25.26	46.38%
WL164	10,695,843	9,940,297	92.90%	180,094	29.59	53.61%
WL165	10,695,843	9,400,558	87.90%	184,653	24.57	48.26%
WL166	9,730,476	8,949,469	92.00%	179,614	21.94	44.03%
WL167	9,730,476	8,937,308	91.80%	181,181	23.68	48.01%
WL168	9,730,476	8,973,347	92.20%	181,327	24.72	49.95%
WL169	9,730,476	8,975,407	92.20%	174,239	25.44	49.39%

WL170	9,730,476	8,468,353	87.00%	182,954	22.63	48.89%
WL171	11,724,737	10,844,298	92.50%	184,952	27.5	46.90%
WL172	11,724,737	10,802,148	92.10%	185,094	27.57	47.24%
WL173	11,724,737	10,849,060	92.50%	185,177	30.22	51.58%
WL174	11,724,737	10,829,518	92.40%	181,077	31.96	53.44%
WL175	11,724,737	10,275,619	87.60%	185,986	25.42	46.01%
WL176	11,182,915	10,411,313	93.10%	184,521	27.69	49.08%
WL177	11,182,915	10,362,341	92.70%	175,588	26.28	44.53%
WL178	11,182,915	10,404,269	93.00%	181,255	26.63	46.39%
WL179	11,182,915	10,382,938	92.80%	181,532	29.57	51.70%
WL180	11,182,915	9,822,318	87.80%	187,040	27.03	51.47%
WL181	10,973,668	10,216,588	93.10%	180,681	25.41	44.94%
WL182	10,973,668	10,145,247	92.50%	177,468	27.24	47.65%
WL183	10,973,668	10,197,334	92.90%	183,116	27.9	50.10%
WL184	10,973,668	10,192,656	92.90%	179,829	28.82	50.85%
WL185	10,973,668	9,627,728	87.70%	183,457	25.45	48.50%
WL186	11,328,008	10,454,244	92.30%	183,810	26.45	46.51%
WL187	11,328,008	10,434,642	92.10%	179,856	28.21	48.62%
WL188	11,328,008	10,452,534	92.30%	182,431	28.88	50.41%
WL189	11,328,008	10,449,617	92.20%	180,707	29.03	50.20%
WL190	11,328,008	9,884,607	87.30%	183,849	25.74	47.88%
WL191	9,608,704	8,916,725	92.80%	178,742	21.82	43.74%
WL192	9,608,704	8,868,255	92.30%	179,947	24.44	49.59%
WL193	9,608,704	8,911,124	92.70%	179,701	23.19	46.76%
WL194	9,608,704	8,899,095	92.60%	177,728	25.29	50.51%
WL195	9,608,704	8,419,925	87.60%	182,904	22.15	48.12%
WL196	10,813,536	10,053,136	93.00%	183,682	24.7	45.13%
WL197	10,813,536	10,009,641	92.60%	182,826	25.79	47.11%
WL198	10,813,536	10,050,389	92.90%	182,207	25.56	46.34%
WL199	10,813,536	10,040,603	92.90%	180,849	26.94	48.52%
WL200	10,813,536	9,518,795	88.00%	186,004	25.51	49.85%
WL201	10,550,973	9,689,941	91.80%	180,997	25.97	48.51%
WL202	10,550,973	9,651,902	91.50%	179,109	26.13	48.49%
WL203	10,550,973	9,696,786	91.90%	177,997	24.75	45.43%
WL204	10,550,973	9,682,736	91.80%	179,014	25.25	46.68%
WL205	10,550,973	9,146,143	86.70%	183,875	24.55	49.36%
WL206	11,751,699	10,803,311	91.90%	183,726	28.44	48.37%
WL207	11,751,699	10,762,605	91.60%	181,916	28.58	48.31%
WL208	11,751,699	10,803,459	91.90%	183,181	29.3	49.68%
WL209	11,751,699	10,802,498	91.90%	181,244	28.73	48.20%
WL210	11,751,699	10,219,567	87.00%	184,922	25.93	46.92%
WL211	10,905,339	10,029,056	92.00%	177,392	25.35	44.84%
WL212	10,905,339	9,994,207	91.60%	179,460	25.86	46.44%
WL213	10,905,339	10,023,513	91.90%	182,325	26.38	47.98%

WL214	10,905,339	10,015,761	91.80%	177,331	26.6	47.10%
WL215	10,905,339	9,442,246	86.60%	181,384	24.75	47.54%
WL216	10,668,845	9,804,622	91.90%	180,479	24.41	44.93%
WL217	10,668,845	9,759,147	91.50%	176,764	24.24	43.91%
WL218	10,668,845	9,788,669	91.80%	182,026	25.58	47.57%
WL219	10,668,845	9,782,066	91.70%	180,052	26.63	49.02%
WL220	10,668,845	9,243,125	86.60%	182,126	24.43	48.14%
WL221	10,488,415	9,687,209	92.40%	181,198	25.18	47.10%
WL222	10,488,415	9,646,086	92.00%	179,045	25.97	48.20%
WL223	10,488,415	9,686,107	92.40%	180,778	25.7	47.97%
WL224	10,488,415	9,680,145	92.30%	180,563	27.17	50.68%
WL225	10,488,415	9,181,040	87.50%	183,511	22.84	45.65%
WL226	10,320,910	9,610,271	93.10%	180,792	24.91	46.86%
WL227	10,320,910	9,580,104	92.80%	178,763	25.02	46.69%
WL228	10,320,910	9,608,045	93.10%	180,192	24.35	45.67%
WL229	10,320,910	9,599,166	93.00%	178,434	26.58	49.41%
WL230	10,320,910	9,114,198	88.30%	182,708	23.43	46.97%
WL231	10,975,079	10,190,581	92.90%	182,435	25.97	46.49%
WL232	10,975,079	10,131,517	92.30%	182,073	27.19	48.86%
WL233	10,975,079	10,171,695	92.70%	183,133	26.71	48.09%
WL234	10,975,079	10,166,403	92.60%	179,393	28.29	49.92%
WL235	10,975,079	9,634,639	87.80%	184,104	24.44	46.70%
WL236	11,480,177	10,613,804	92.50%	183,519	28.17	48.71%
WL237	11,480,177	10,572,899	92.10%	180,824	27.55	47.12%
WL238	11,480,177	10,618,338	92.50%	180,174	24.62	41.78%
WL239	11,480,177	10,594,162	92.30%	182,457	29.51	50.82%
WL240	11,480,177	10,044,599	87.50%	185,124	25.7	47.37%
WL241	11,701,093	10,837,363	92.60%	183,760	26.69	45.26%
WL242	11,701,093	10,765,337	92.00%	184,322	29.23	50.05%
WL243	11,701,093	10,805,626	92.30%	185,753	29.31	50.39%
WL244	11,701,093	10,794,499	92.30%	182,114	29.11	49.11%
WL245	11,701,093	10,213,082	87.30%	185,929	25.68	46.75%
WL246	13,282,430	12,362,758	93.10%	184,236	29.81	44.42%
WL247	13,282,430	12,309,574	92.70%	184,279	29.94	44.82%
WL248	13,282,430	12,332,983	92.90%	187,618	33.37	50.76%
WL249	13,282,430	12,323,867	92.80%	184,661	35.28	52.86%
WL250	13,282,430	11,705,046	88.10%	187,526	30.67	49.14%
WL251	10,704,846	9,904,767	92.50%	184,001	25.33	47.06%
WL252	10,704,846	9,859,114	92.10%	178,307	26.84	48.54%
WL253	10,704,846	9,908,926	92.60%	182,477	27	49.72%
WL254	10,704,846	9,898,919	92.50%	178,632	29.25	52.78%
WL255	10,704,846	9,372,299	87.60%	184,466	25.67	50.52%
WL256	10,197,915	9,683,244	95.00%	182,156	25.37	47.72%
WL257	10,197,915	9,662,193	94.70%	176,594	25	45.69%

WL258	10,197,915	9,694,079	95.10%	181,144	25.61	47.85%
WL259	10,197,915	9,703,621	95.20%	176,834	24.75	45.10%
WL260	10,197,915	9,618,599	94.30%	186,440	25.74	49.89%
WL261	13,600,004	12,976,152	95.40%	182,774	32.9	46.34%
WL262	13,600,004	12,963,306	95.30%	179,013	31.72	43.80%
WL263	13,600,004	12,982,288	95.50%	183,095	30.59	43.14%
WL264	13,600,004	12,983,109	95.50%	181,659	33.55	46.94%
WL265	13,600,004	12,885,681	94.70%	186,999	33.27	48.28%
WL266	13,002,100	12,239,397	94.10%	183,188	32.01	47.91%
WL267	13,002,100	12,223,868	94.00%	180,257	29.59	43.63%
WL268	13,002,100	12,243,498	94.20%	185,406	31.79	48.14%
WL269	13,002,100	12,261,428	94.30%	181,460	30.95	45.80%
WL270	13,002,100	12,146,062	93.40%	185,902	29.54	45.21%
WL271	10,625,864	10,100,222	95.10%	173,178	27.57	47.27%
WL272	10,625,864	10,090,822	95.00%	178,429	26.43	46.73%
WL273	10,625,864	10,117,212	95.20%	180,485	25.32	45.17%
WL274	10,625,864	10,117,440	95.20%	177,817	27.59	48.49%
WL275	10,625,864	10,054,037	94.60%	184,709	25.59	47.01%
WL276	11,702,004	11,104,211	94.90%	181,422	28.49	46.55%
WL277	11,702,004	11,090,953	94.80%	178,342	29.81	47.93%
WL278	11,702,004	11,115,083	95.00%	183,798	29.64	49.01%
WL279	11,702,004	11,105,318	94.90%	178,589	29.61	47.62%
WL280	11,702,004	11,037,303	94.30%	186,228	29.2	49.27%
WL281	10,892,305	10,325,427	94.80%	180,308	26.24	45.82%
WL282	10,892,305	10,310,646	94.70%	177,713	24.88	42.88%
WL283	10,892,305	10,329,498	94.80%	181,286	28.27	49.61%
WL284	10,892,305	10,333,168	94.90%	176,885	26.77	45.83%
WL285	10,892,305	10,262,727	94.20%	185,658	27.31	49.41%
WL286	15,635,294	14,749,482	94.30%	185,221	40.25	50.55%
WL287	15,635,294	14,716,754	94.10%	184,031	38.11	47.66%
WL288	15,635,294	14,786,615	94.60%	186,765	35.88	45.32%
WL289	15,635,294	14,769,561	94.50%	186,169	39.21	49.42%
WL290	15,635,294	14,663,639	93.80%	188,903	36.68	47.25%
WL291	12,125,418	11,464,931	94.60%	182,443	26.42	42.04%
WL292	12,125,418	11,450,431	94.40%	183,600	28.26	45.31%
WL293	12,125,418	11,475,380	94.60%	186,050	29.02	47.05%
WL294	12,125,418	11,478,808	94.70%	183,494	32.46	51.89%
WL295	12,125,418	11,395,549	94.00%	187,331	29.11	47.85%
WL296	11,592,815	10,933,400	94.30%	183,795	28.85	48.50%
WL297	11,592,815	10,939,357	94.40%	180,831	25.51	42.17%
WL298	11,592,815	10,964,686	94.60%	186,394	29.71	50.51%
WL299	11,592,815	10,961,687	94.60%	183,060	30.47	50.88%
WL300	11,592,815	10,882,364	93.90%	185,589	27.19	46.37%
WL301	22,925,677	21,785,846	95.00%	189,128	49.43	42.91%

WL302	22,925,677	21,753,918	94.90%	188,991	55.13	47.90%
WL303	22,925,677	21,833,052	95.20%	190,674	55.1	48.12%
WL304	22,925,677	21,834,282	95.20%	189,925	58.65	51.02%
WL305	22,925,677	21,706,522	94.70%	190,659	54.04	47.47%
WL306	39,503,519	35,036,511	88.70%	180,589	88.55	45.64%
WL307	39,503,519	35,673,394	90.30%	181,824	92.01	46.90%
WL308	39,503,519	34,926,246	88.40%	190,353	88.96	48.48%

Table S4 Description of characteristics of the 20 linkage groups in the high-density genetic map.

Chr.	No. of SNP in map	Map length (cM)	Average interval (cM)
Chr1	137	93.6	0.69
Chr2	117	147.89	1.27
Chr3	260	126	0.49
Chr4	124	120.63	0.98
Chr5	152	126.63	0.84
Chr6	186	134.45	0.73
Chr7	146	111.11	0.77
Chr8	252	163.85	0.65
Chr9	230	110.48	0.48
Chr10	187	133.83	0.72
Chr11	70	137.42	1.99
Chr12	72	107.2	1.51
Chr13	185	130.61	0.71
Chr14	202	110.97	0.55
Chr15	182	72.62	0.4
Chr16	88	95.28	1.1
Chr17	258	131.24	0.51
Chr18	220	105.52	0.48
Chr19	237	133.37	0.57
Chr20	149	117.96	0.8
Sum	3454	2208.16	0.64

Table S5 Putative QTLs for soybean flowering time identified using an RIL population grown in eight different environments and using BLUP values.

QTL[a]	Marker interval	Range(cM)	Size(Mb)	Env.[b]	*A*[c]	PVE[d](%)	LOD	Reported Gene
qFT5-1	Chr05-29831567-Chr05-32921731	12.47	3.09	XX16	1.20	1.39	5.65	
qFT5-2	Chr05-33027202-Chr05-33140934	1.17	0.11	XT17	1.46	2.25	5.97	
qFT5-3	Chr05-34388361-Chr05-34842094	4.28	0.45	XX17	1.41	1.24	4.35	
				BJ17	1.73	1.17	4.91	
				BLUP	0.96	1.18	6.06	
qFT6	Chr06-15974931-Chr06-23215177	9.43	7.24	SY16	1.74	30.26	44.44	*E1*, ref. 1
				XX16	5.91	27.09	67.10	
				JN16	5.50	21.82	52.55	
				BJ16	8.81	29.50	67.99	
				SY17	0.62	13.39	14.82	
				XT17	5.09	22.43	44.05	
				XX17	6.76	23.19	51.96	
				BJ17	9.42	27.86	68.13	
				BLUP	5.18	27.81	77.55	
qFT10	Chr10-44578784-Chr10-46195658	8.73	1.62	SY16	0.86	7.48	13.09	*E2*, ref. 2
				XX16	3.88	12.28	37.73	
				JN16	4.42	14.37	38.97	
				BJ16	5.35	11.34	34.49	
				XT17	3.70	11.97	26.23	
				XX17	5.07	13.55	35.74	
				BJ17	6.63	14.40	43.36	
				BLUP	3.69	14.42	49.84	
qFT11	Chr11-10144020-Chr11-11875877	3.23	0.46	XX16	1.70	2.78	10.46	
				JN16	2.26	4.51	9.64	
				BJ16	1.63	1.26	5.00	
				XX17	2.95	5.44	10.65	
				BJ17	2.95	3.42	12.86	
				BLUP	1.48	2.77	8.13	
qFT12-1	Chr12-1839081-Chr12-2611000	3.24	0.77	XX16	1.04	1.05	4.34	
				JN16	1.08	1.04	3.86	
				BLUP	0.73	0.69	3.57	
qFT12-2	Chr12-5445349-Chr12-6081748	3.78	0.64	XX16	3.34	10.86	35.82	

				JN16	3.11	8.64	26.86	
				BJ16	4.22	8.48	28.53	
				SY17	0.32	4.47	5.37	
				XT17	2.86	8.57	20.59	
				XX17	4.93	15.40	40.11	
				BJ17	5.33	11.20	36.45	
				BLUP	2.63	8.89	35.94	
qFT15		8.75	9.14	XT17	1.13	1.35	3.30	
qFT16-1	Chr16-3664326-Chr16-4720071	8.05	1.06	SY16	0.65	5.04	9.47	*GmFT5a*, ref. 3, ref.4
				XX16	4.07	15.93	44.36	
				JN16	4.69	19.45	46.00	
				BJ16	6.52	20.07	51.04	
				SY17	0.26	2.90	3.51	
				XT17	3.43	12.23	25.53	
				XX17	4.19	11.05	30.63	
				BJ17	6.75	17.84	47.97	
				BLUP	3.82	18.65	57.13	
qFT16-2	Chr16-30470481-Chr16-33434040	21.36	2.96	SY16	1.24	18.66	23.82	*GmFT2a*, ref. 3, ref.5
				XX16	2.22	4.67	11.58	
				JN16	2.32	4.74	11.48	
				BJ16	2.78	3.61	8.65	
				SY17	0.55	12.82	10.88	
				XT17	1.76	3.20	5.97	
				XX17	1.90	2.24	5.24	
				BJ17	2.21	1.88	5.18	
				BLUP	1.87	4.39	13.31	
qFT16-3	Chr16-4444619-Chr16-4720071	0.86	0.28	SY17	0.26	2.90	3.51	
qFT17	Chr17-6240391-Chr17-6353202	2.63	0.11	XT17	1.33	1.88	5.07	
qFT18	Chr18-52438025-Chr18-52419931	0.02	0.02	SY17	0.29	3.71	4.54	
qFT19	Chr19-47586598-Chr19-49013328	14.682	1.43	SY16	0.43	2.28	4.62	*E3*, ref. 6
				XX16	3.35	10.87	35.58	
				JN16	2.88	7.39	23.59	
				BJ16	4.39	9.14	27.88	
				XT17	3.80	15.15	32.77	
				XX17	3.78	9.06	23.19	
				BJ17	4.50	7.98	26.04	
				BLUP	2.65	9.00	33.71	

[a]The name of QTL, is a composite of the influenced trait: FT (flowering time), followed by the chromosome number

[b]Env., represents environment; SY16, XX16, JN16 and BJ16 represent Sanya, Xinxiang, Jining and Beijing in 2016, respectively; SY17, XT17, XX17 and BJ17 represent Sanya, Xiangtan, Xinxiang and Beijing in 2017, respectively

[c]The additive effect of the QTL

[d]The percentage of phenotypic variation explained by corresponding QTL

Ref. 1 Xia, Z., Watanabe, S., Yamada, T., Tsubokura, Y., Nakashima, H., Zhai, H., Anai, T., Sato, S., Yamazaki, T., Lu, S., Wu, H., Tabata, S. and Harada, K. (2012) Positional cloning and characterization reveal the molecular basis for soybean maturity locus *E1* that regulates photoperiodic flowering. *Proc. Natl Acad. Sci. USA*, **109**, E2155-2164.

Ref. 2 Watanabe, S., Xia, Z., Hideshima, R., Tsubokura, Y., Sato, S., Yamanaka, N., Takahashi, R., Anai, T., Tabata, S., Kitamura, K. and Harada, K. (2011) A map-based cloning strategy employing a residual heterozygous line reveals that the *GIGANTEA* gene is involved in soybean maturity and flowering. *Genetics*, **188**, 395-407.

Ref. 3 Kong, F., Liu, B., Xia, Z., Sato, S., Kim, B.M., Watanabe, S., Yamada, T., Tabata, S., Kanazawa, A., Harada, K. and Abe, J. (2010) Two coordinately regulated homologs of *FLOWERING LOCUS T* are involved in the control of photoperiodic flowering in soybean. *Plant Physiol.* **154**, 1220-1231.

Ref. 4 Takeshima, R., Hayashi, T., Zhu, J., Zhao, C., Xu, M., Yamaguchi, N., Sayama, T., Ishimoto, M., Kong, L., Shi, X., Liu, B., Tian, Z., Yamada, T., Kong, F. and Abe, J. (2016) A soybean quantitative trait locus that promotes flowering under long days is identified as *FT5a*, a *FLOWERING LOCUS T* ortholog. *J. Exp. Bot.* **67**, 5247-5258.

Ref. 5 Kong, F., Nan, H., Cao, D., Li, Y., Wu, F., Wang, J., Lu, S., Yuan, X., Cober, E.R. and Abe, J. (2014) A new dominant gene *E9* conditions early flowering and maturity in soybean. *Crop Sci.* **54**, 2529-2535.

Ref. 6 Watanabe, S., Hideshima, R., Xia, Z., Tsubokura, Y., Sato, S., Nakamoto, Y., Yamanaka, N., Takahashi, R., Ishimoto, M., Anai, T., Tabata, S. and Harada, K. (2009) Map-based cloning of the gene associated with the soybean maturity locus *E3*. *Genetics*, **182**, 1251-1262.

Table S6 Predicted genes located in the mapped 636-kb genomic region of *qFT12-2* in the Williams 82 reference genome.

Gene Number	Phytozome ID	Location	GO	Othologues in Arabidopsis
1	Glyma.12G073400	5468987-5469193	There are no functional annotations for this locus	-
2	Glyma.12G073500	5476826-5477457	There are no functional annotations for this locus	-
3	Glyma.12G073600	5488931-5490506	There are no functional annotations for this locus	-
4	Glyma.12G073700	5497564-5501763	Protein kinase superfamily protein	ATMPK13
5	Glyma.12G073800	5507147-5508540	There are no functional annotations for this locus	-
6	Glyma.12G073900	5508365-5522772	pseudo-response regulator 7	APRR7,PRR7
7	Glyma.12G074000	5534233-5538664	Protein kinase superfamily protein	-
8	Glyma.12G074100	5543485-5564378	phototropin 1	JK224,NPH1,PHOT1,RPT1
9	Glyma.12G074200	5567749-5570710	BTB/POZ domain-containing protein	-
10	Glyma.12G074300	5572236-5577945	P-loop containing nucleoside triphosphate hydrolases superfamily protein	-
11	Glyma.12G074400	5601542-5604778	CCT motif family protein	-
12	Glyma.12G074500	5616463-5617266	Histone superfamily protein	-
13	Glyma.12G074600	5628265-5632199	hercules receptor kinase 1	HERK1
14	Glyma.12G074700	5637860-5645670	NOT2 / NOT3 / NOT5 family	-
15	Glyma.12G074800	5646423-5654810	There are no functional annotations for this locus	-
16	Glyma.12G074900	5658150-5662813	Ankyrin repeat family protein	-
17	Glyma.12G075000	5674766-5680512	nudix hydrolase homolog 9	aTNUDT9,NUDT9
18	Glyma.12G075100	5692181-5694308	3-ketoacyl-CoA synthase 12	KCS12

19	Glyma.12G075200	5723240-5725084	exocyst subunit exo70 family protein H7	ATEXO70H7,EXO70H7
20	Glyma.12G075300	5739446-5741060	Heavy metal transport/detoxification superfamily protein	-
21	Glyma.12G075400	5747587-5750039	plasma membrane intrinsic protein 2,4	PIP2,4,PIP2F
22	Glyma.12G075500	5768615-5770637	Ribosomal protein L11 family protein	-
23	Glyma.12G075600	5776807-5778036	Protein of unknown function, DUF584	-
24	Glyma.12G075700	5794863-5798468	dsRNA-binding protein 2	DRB2
25	Glyma.12G075800	5813285-5820142	Homeobox-leucine zipper family protein / lipid-binding START domain-containing protein	IFL,IFL1,REV
26	Glyma.12G075900	5822268-5826790	Uncharacterised protein family (UPF0497)	-
27	Glyma.12G076000	5828539-5829613	There are no functional annotations for this locus	-
28	Glyma.12G076100	5833380-5842798	SIT4 phosphatase-associated family protein	-
29	Glyma.12G076200	5851956-5855787	auxin response factor 10	ARF10
30	Glyma.12G076300	5858282-5858821	There are no functional annotations for this locus	-
31	Glyma.12G076400	5860161-5860865	There are no functional annotations for this locus	-
32	Glyma.12G076500	5869107-5875332	GATA transcription factor 11	GATA11
33	Glyma.12G076600	5876603-5881823	glycosyltransferase family protein 2	-
34	Glyma.12G076700	5885738-5891879	Putative lysine decarboxylase family protein	ATLOG1,LOG1
35	Glyma.12G076800	5912502-5919345	cyclic nucleotide-gated channel 15	ATCNGC15,CNGC15
36	Glyma.12G076900	5934441-5939599	3\'-5\'-exoribonuclease family protein	-
37	Glyma.12G077000	5934761-5935478	There are no functional annotations for this locus	-
38	Glyma.12G077100	5940924-5942542	senescence-associated gene 12	SAG12

39	Glyma.12G077200	5951472-5953373	senescence-associated gene 12	SAG13
40	Glyma.12G077300	5957770-5964375	Protein kinase superfamily protein	NCRK
41	Glyma.12G077400	6009344-6022524	ATP-dependent helicase family protein	MOM,MOM1
42	Glyma.12G077500	6021651-6025927	protein kinase family protein / peptidoglycan-binding LysM domain-containing protein	-
43	Glyma.12G077600	6031677-6032659	There are no functional annotations for this locus	-
44	Glyma.12G077700	6036093-6037146	There are no functional annotations for this locus	-
45	Glyma.12G077800	6041826-6049255	BEL1-like homeodomain 8	BLH8,PNF
46	Glyma.12G077900	6062065-6068997	Protein of unknown function, DUF547	-
47	Glyma.12G078000	6072988-6079965	ABC transporter family protein	-

Table S7 Haplotypes of *GmPRR37* in 180 soybean cultivars from China.

**Cultivar**	**Region**	**Source**	***GmPRR37***
Heilongjiang41	Northern China	Heilongjiang	*Gmprr37*
Kexi283	Northern China	Heilongjiang	*Gmprr37*
Heihe3	Northern China	Heilongjiang	*Gmprr37*
Fengshou10	Northern China	Heilongjiang	*Gmprr37*
Heihe51	Northern China	Heilongjiang	*Gmprr37*
Fengshou12	Northern China	Heilongjiang	*Gmprr37*
Fengshou17	Northern China	Heilongjiang	*Gmprr37*
Beifeng2	Northern China	Heilongjiang	*Gmprr37*
Fengshou19	Northern China	Heilongjiang	*Gmprr37*
Heihe9	Northern China	Heilongjiang	*Gmprr37*
Beifeng9	Northern China	Heilongjiang	*Gmprr37*
Heihe19	Northern China	Heilongjiang	*Gmprr37*
Heihe27	Northern China	Heilongjiang	*Gmprr37*
Fengshou24	Northern China	Heilongjiang	*Gmprr37*
Heihe38	Northern China	Heilongjiang	*Gmprr37*
Beidou5	Northern China	Heilongjiang	*Gmprr37*
Jiangmodou1	Northern China	Heilongjiang	*Gmprr37*
Huajiang4	Northern China	Heilongjiang	*Gmprr37*
Dengke1	Northern China	Heilongjiang	*Gmprr37*
Mengdou30	Northern China	Neimenggu	*Gmprr37*
Beifeng11	Northern China	Heilongjiang	*Gmprr37*
Heihe18	Northern China	Heilongjiang	*Gmprr37*
Jinyuan2	Northern China	Heilongjiang	*Gmprr37*
Mancangjin	Northern China	Heilongjiang	*Gmprr37*
Jingshanpu	Northern China	Heilongjiang	*Gmprr37*
Zhi2	Northern China	Jilin	*Gmprr37*
Dongnong4	Northern China	Heilongjiang	*Gmprr37*
Hejiao8	Northern China	Heilongjiang	*Gmprr37*
Hejiao6	Northern China	Heilongjiang	*Gmprr37*
Heinong16	Northern China	Heilongjiang	*Gmprr37*
Suinong3	Northern China	Heilongjiang	*Gmprr37*
Hefeng22	Northern China	Heilongjiang	*Gmprr37*
Heinong26	Northern China	Heilongjiang	*Gmprr37*
Hefeng25	Northern China	Heilongjiang	*Gmprr37*
Heinong33	Northern China	Heilongjiang	*Gmprr37*
Suinong8	Northern China	Heilongjiang	*Gmprr37*
Heinong35	Northern China	Heilongjiang	*Gmprr37*
Suinong10	Northern China	Heilongjiang	*Gmprr37*
Hefeng35	Northern China	Heilongjiang	*Gmprr37*
Suinong41	Northern China	Heilongjiang	*Gmprr37*
Heinong43	Northern China	Heilongjiang	*Gmprr37*
Heinong44	Northern China	Heilongjiang	*Gmprr37*

Hefeng45	Northern China	Heilongjiang	*Gmprr37*
Hefeng50	Northern China	Heilongjiang	*Gmprr37*
Hefeng47	Northern China	Heilongjiang	*Gmprr37*
Suinong28	Northern China	Heilongjiang	*Gmprr37*
Kenfeng16	Northern China	Heilongjiang	*Gmprr37*
Heinong48	Northern China	Heilongjiang	*Gmprr37*
Hefeng55	Northern China	Heilongjiang	*Gmprr37*
Kangxian4	Northern China	Heilongjiang	*Gmprr37*
Heinong37	Northern China	Heilongjiang	*Gmprr37*
Liaodou15	Northern China	Liaoning	*Gmprr37*
Fushou	Northern China	Neimenggu	*Gmprr37*
Huangbaozhu	Northern China	Jilin	*Gmprr37*
Xiaojinhuang1	Northern China	Jilin	*Gmprr37*
FengdiHuang	Northern China	Jilin	*Gmprr37*
Jiti1	Northern China	Liaoning	*Gmprr37*
Jiti5	Northern China	Jilin	*Gmprr37*
Jilin3	Northern China	Jilin	*Gmprr37*
Jilin6	Northern China	Jilin	*Gmprr37*
Tiefeng3	Northern China	Liaoning	*Gmprr37*
Tiefeng18	Northern China	Liaoning	*Gmprr37*
Kaiyu3	Northern China	Liaoning	*Gmprr37*
Jilin13	Northern China	Jilin	*Gmprr37*
Jiunong9	Northern China	Jilin	*Gmprr37*
Kaiyu8	Northern China	Liaoning	*Gmprr37*
Changnong4	Northern China	Jilin	*Gmprr37*
Jilin20	Northern China	Jilin	*Gmprr37*
Kaiyu10	Northern China	Liaoning	*Gmprr37*
Changnong5	Northern China	Jilin	*Gmprr37*
Jilin30	Northern China	Jilin	*Gmprr37*
Tiefeng29	Northern China	Liaoning	*Gmprr37*
Jiunong22	Northern China	Jilin	*Gmprr37*
Jilin47	Northern China	Jilin	*Gmprr37*
Tiefeng31	Northern China	Liaoning	*Gmprr37*
Jiyu57	Northern China	Jilin	*Gmprr37*
Zhonghuang35	Yellow-Huai-Hai	Hebei	*Gmprr37*
Haiyangpamanqing	Yellow-Huai-Hai	Shandong	*Gmprr37*
Shangcaiercaoshipingdingshi	Yellow-Huai-Hai	Henan	*Gmprr37*
Hezeniumaohuang	Yellow-Huai-Hai	Shandong	*Gmprr37*
Yidupingdinghuang	Yellow-Huai-Hai	Shandong	*Gmprr37*
Qihuang10	Yellow-Huai-Hai	Shandong	*Gmprr37*
Yanhuang1	Yellow-Huai-Hai	Shandong	*Gmprr37*
Henanzaofeng1	Yellow-Huai-Hai	Henan	*Gmprr37*
Wenfeng7	Yellow-Huai-Hai	Shandong	*Gmprr37*
Yuejin5	Yellow-Huai-Hai	Shandong	*Gmprr37*

Youbian30	Yellow-Huai-Hai	Hebei	*Gmprr37*
Ludou4	Yellow-Huai-Hai	Shandong	*Gmprr37*
Yudou2	Yellow-Huai-Hai	Henan	*Gmprr37*
Zhongdou19	Yellow-Huai-Hai	Hubei	*Gmprr37*
Kefeng6	Yellow-Huai-Hai	Hebei	*Gmprr37*
Jidou7	Yellow-Huai-Hai	Hebei	*Gmprr37*
Ludou11	Yellow-Huai-Hai	Shandong	*Gmprr37*
Jidou12	Yellow-Huai-Hai	Hebei	*Gmprr37*
Yudou22	Yellow-Huai-Hai	Henan	*Gmprr37*
Zhonghuang13	Yellow-Huai-Hai	Hebei	*Gmprr37*
Zheng92116	Yellow-Huai-Hai	Henan	*Gmprr37*
Handou5	Yellow-Huai-Hai	Hebei	*Gmprr37*
Yudou8	Yellow-Huai-Hai	Henan	*Gmprr37*
Xudou9	Yellow-Huai-Hai	Jiangsu	*Gmprr37*
Hedou13	Yellow-Huai-Hai	Shandong	*Gmprr37*
Jidou17	Yellow-Huai-Hai	Hebei	*Gmprr37*
Wandou24	Yellow-Huai-Hai	Aahui	*Gmprr37*
Zhonghuang37	Yellow-Huai-Hai	Hebei	*Gmprr37*
Zhonghuang30	Yellow-Huai-Hai	Hebei	*Gmprr37*
Jindou19	Yellow-Huai-Hai	Shanxi	*Gmprr37*
Jindou21	Yellow-Huai-Hai	Shanxi	*Gmprr37*
Jindou23	Yellow-Huai-Hai	Shanxi	*Gmprr37*
Jindou25	Yellow-Huai-Hai	Shanxi	*Gmprr37*
Aijiaozao	Southern China	Hubei	*Gmprr37*
Zhechun3	Southern China	Zhejiang	*Gmprr37*
Guichun1	Southern China	Guangxi	*Gmprr37*
Guichun8	Southern China	Guangxi	*Gmprr37*
Tianlong1	Southern China	Hubei	*Gmprr37*
Houzimao	Southern China	Hubei	*Gmprr37*
Edou8	Southern China	Hubei	*Gmprr37*
Dian86-4	Southern China	Yunnan	*Gmprr37*
Jinningdahuangdou	Southern China	Yunnan	*Gmprr37*
Qiandou6	Southern China	Guizhou	*Gmprr37*
Taiwan75	Southern China	Taiwan	*Gmprr37*
Suxie1	Southern China	Jiangsu	*Gmprr37*
Zihua4	Northern China	Heilongjiang	*Gmprr37*
Jingda332	Southern China	Jiangsu	*Gmprr37*
Beihudou	Northern China	Heilongjiang	*Gmprr37*
Heihe54	Northern China	Heilongjiang	*Gmprr37*
Dongnong72-806	Northern China	Heilongjiang	*Gmprr37*
Taixingheidou	Southern China	Jiangsu	*Gmprr37*
Miquanhuangdou	Northern China	Xinjiang	*Gmprr37*
Changjihuangdou	Northern China	Xinjiang	*Gmprr37*
Jindou3	Northern China	Shanxi	*Gmprr37*

Jilin4	Northern China	Jilin	*Gmprr37*
Zaofeng1	Northern China	Jilin	*Gmprr37*
Qunyingdou	Yellow-Huai-Hai	Hebei	*Gmprr37*
Jindou1	Northern China	Shanxi	*Gmprr37*
Tiefeng20	Northern China	Liaoning	*Gmprr37*
Tiefeng19	Northern China	Liaoning	*Gmprr37*
Jin6604-24	Northern China	Liaoning	*Gmprr37*
Jing33	Northern China	Liaoning	*Gmprr37*
Jindou2	Northern China	Shanxi	*Gmprr37*
Tiefeng8	Northern China	Liaoning	*Gmprr37*
Dandou4	Northern China	Liaoning	*Gmprr37*
Jing8-14	Northern China	Liaoning	*Gmprr37*
Wenfeng5	Yellow-Huai-Hai	Shandong	*Gmprr37*
Zhengzhou135	Yellow-Huai-Hai	Henan	*Gmprr37*
Weiqingdou	Yellow-Huai-Hai	Henan	*Gmprr37*
Shangyukanshanbai	Southern China	Zhejiang	*Gmprr37*
Fengchengniupidou	Southern China	Jiangxi	*Gmprr37*
Dandou1	Northern China	Liaoning	*Gmprr37*
Changpingqingdou	Northern China	Beijing	*Gmprr37*
Huairouhuangdou	Yellow-Huai-Hai	Beijing	*Gmprr37*
Xudou1	Yellow-Huai-Hai	Jiangsu	*Gmprr37*
Xudou2	Yellow-Huai-Hai	Jiangsu	*Gmprr37*
Xudou5	Yellow-Huai-Hai	Jiangsu	*Gmprr37*
Yuejing4	Yellow-Huai-Hai	Shandong	*Gmprr37*
Hai94	Yellow-Huai-Hai	Shanxi	*Gmprr37*
Yunyizao	Southern China	Hunan	*Gmprr37*
Chengliuniumaohuang	Southern China	Henan	*Gmprr37*
Liuyuehuang	Southern China	Guizhou	*Gmprr37*
Yangchunqingdou	Southern China	Guangdong	*Gmprr37*
Baihuadou	Southern China	Guangdong	*Gmprr37*
Jinjiangdaqingren	Southern China	Fujian	*Gmprr37*
58-161	Yellow-Huai-Hai	Jiangsu	*Gmprr37*
Yulingdahuangdou	Southern China	Guangxi	*Gmprr37*
Juhuangdadou	Southern China	Guangdong	*Gmprr37*
Edou2	Southern China	Hubei	*Gmprr37*
Wuhuasiyuehuang	Southern China	Guangdong	*Gmprr37*
Zigongqingpidou	Southern China	Sichuan	*Gmprr37*
Suidaohuang	Southern China	Jiangsu	*Gmprr37*
Nannong493-1	Southern China	Jiangsu	*Gmprr37*
Pingguohuangdou	Southern China	Guangxi	*Gmprr37*
Lanxidaqingdou	Southern China	Zhejiang	*Gmprr37*
Bayueqing	Southern China	Hunan	*Gmprr37*
Dandou2	Northern China	Liaoning	*GmPRR37-1*
Zigongdongdou	Southern China	Sichuan	*GmPRR37-1*

Bahong1	Yellow-Huai-Hai	Hebei	*GmPRR37-1*
Nandou12	Southern China	Sichuan	*GmPRR37-2*
Heibiqing	Southern China	Guangdong	*GmPRR37-2*
Naiyingheidou	Yellow-Huai-Hai	Hebei	*GmPRR37-2*
Shangraodaqingsi	Southern China	Jiangxi	*GmPRR37-2*
Qiudou1	Southern China	Hunan	*GmPRR37-2*

Table S8 Primer sequences used in this study.

Primer name	Sequence (5'→3')	Application
WPP-F	TCCTGACCTCAAGTCCAGTAT	For *GmPRR37* genomic sequence cloning
WPP-R	CACAACATGGCGTGTCGAAT	
WP1-F	TGGCTCTTCCATTTTTATACCTGT	
WP1-R	TGGGACCTCCCTAGAGGATAC	
WP2-F	TGGGCTCCATGTTTTATGCATTTAT	
WP2-R	GCTCCAAAGACCAACTGGTAGA	
WP3-F	ACGAACCCAACTTGTCACGAAT	
WP3-R	GAGCCAGGTAACTTTAGGCTTT	
WP4-F	TATCACAAGGGGCACGGAAT	
WP4-R	GCAAAAACAGTGTCCGACCATT	
WP5-F	AGCTTCTGTGTCACCCTTTAATCA	
WP5-R	GACAAAATCCACATGCAACAAAGA	
WP6-F	CATTGGGAACGGGACAGATG	
WP6-R	ATTTTACTAGAAGAGGGGGCCAT	
GmPRR37-F	ATGAACAATAATGTTGGGAAAGGGA	For *GmPRR37* CDS cloning
GmPRR37-R	TTGAGGAATGTCGATAGAATTGTCC	
qGmPRR37-F	GAAAGTGCGACCCTCACCAG	For Real-Time Quantitative PCR
qGmPRR37-R	TCTGAGTGCCACTTCCATTGT	
qGmELF3-F	ACAGGGATTAAGCACTGGGC	
qGmELF3-R	TGCAGTTGCAGATCTTCGGT	
qGmGIa-F	GCTCTGCCTCAAAAAACCTGGCCG	
qGmGIa-R	GAAGCTAAATGATTATTCCCCCG	
qGmCOL1a-F	TCAAAAGCGCTAGTGTCCCT	
qGmCOL1a-R	AAAGTGGTGGTTGTCGCAGT	
qGmCOLb-F	GAGTGCTACTGTCCCTAACACCAA	
qGmCOLb-R	CCAGCGAAATGTTGGTGCTGAGC	
qGmFT1a-F	CCCAGCAAGGTAATCTGCAA	
qGmFT1a-R	ATCGATTATTTCCTCATACGTACA	
qGmFT2a-F	ATGCACCTAGCCCAAGTGAC	
qGmFT2a-R	TACACGGTCTCCCTACCCAG	
qGmFT5a-F	CACGGGAGAACCCTCTTGTTAT	
qGmFT5a-R	GGTCTTCACCACCAACAGTAACC	
qGmActin-F	CGGTGGTTCTATCTTGGCATC	
qGmActin-R	GTCTTTCGCTTCAATAACCCTA	
*Hind*III-*GmPRR37-F*	CAAATCGACTCTAGAAAGCTTATGAACAATAATGTTGGGAAAGGGA	For construction of 35S::*GmPRR37-GFP*
*Spe*I-*GmPRR37-R*	CATGGTACCGGATCCACTAGTTTGAGGAATGTCGATAGAATTGTCC	
OE-GmPRR37-F	CTAGCAGAACAGCGACCACG	For transgenic plants checking
OE-GmPRR37-R	AAGAAGATGGTGCGCTCCTG	
KGmPRR37-F1	GAAGGTGACCAAGTTCATGCTTCAGTAAGAGATCATGATCTGATTA	For genotyping of *GmPRR37* using KASP

KGmPRR37-F2	GAAGGTCGGAGTCAACGGATTTCAGT<br>AAGAGATCATGATCTGATTG
KGmPRR37-R	AGAATCAGTAGGAGGCTCTGAACAA
